# Supplementary material for: Tamoxifen for the treatment of myeloproliferative neoplasms: A Phase II clinical trial and exploratory analysis
Source: Nat Commun. 2023 Nov 25;14:7725. doi: 10.1038/s41467-023-43175-5 (PMC10673935; doi:10.1038/s41467-023-43175-5)
Supplement: Supplementary file 1 — Supplementary Information [file 41467_2023_43175_MOESM1_ESM.pdf]

Supplementary Information

Title: Tamoxifen for the treatment of myeloproliferative neoplasms: A Phase II clinical trial and exploratory analysis

Author list:

Zijian Fang[1-3], Giuditta Corbizi Fattori[1-3, #], Thomas McKerrell[1-3,5, #], Rebecca H Boucher[4], Aimee Jackson[4], Rachel S Fletcher[4], Dorian Forte[1-3], Jose-Ezequiel Martin[6], Sonia Fox[4], James Roberts[2], Rachel Glover[2], Erica Harris[2], Hannah R Bridges[7], Luigi Grassi[2], Alba Rodriguez-Meira[8], Adam J Mead[8], Steven Knapper[9], Joanne Ewing[10], Nauman M Butt[11], Manish Jain[12], Sebastian Francis[13], Fiona J Clark[10], Jason Coppell[14], Mary F McMullin[15], Frances Wadelin[16],Srinivasan Narayanan[17], Dragana Milojkovic[18], Mark W Drummond[19], Mallika Sekhar[20], Hesham ElDaly[5], Judy Hirst[7], Maike Paramor[1], E Joanna Baxter[2], Anna L Godfrey[5], Claire N Harrison[21,]* , Simón Méndez-Ferrer[1-3,]*

Affiliations:

[1]Wellcome-MRC Cambridge Stem Cell Institute, Cambridge, UK.
[2]Department of Haematology, University of Cambridge, Cambridge, UK.
[3]NHS Blood and Transplant, Cambridge, UK.
[4]University of Birmingham, Birmingham, UK.
[5]Cambridge University Hospitals NHS Foundation Trust, Cambridge, UK.
[6]Cancer Molecular Diagnostic Laboratory, Department of Oncology, University of Cambridge, Cambridge, UK. [7]MRC Mitochondrial Biology Unit, University of Cambridge, Cambridge, UK.
[8]NIHR Biomedical Research Centre and MRC Molecular Haematology Unit, Weatherall Institute of Molecular Medicine, University of Oxford, Oxford, UK.
[9]Cardiff University, Cardiff, UK.
[10]University Hospitals Birmingham NHS Foundation Trust, Birmingham, UK.
[11]The Clatterbridge Cancer Centre NHS Foundation Trust, Liverpool, UK.
[12]St James University Hospital, Leeds, UK.
[13]Sheffield Teaching Hospitals NHS Trust, Sheffield, UK.
[14]Royal Devon and Exeter Hospital, Exeter, UK.
[15]Queens University, Belfast, UK.
[16]Nottingham University Hospital, Nottingham, UK.
[17]University Hospital Southampton NHSFT, Southampton, UK.
[18]Imperial College Healthcare NHS Trust, London, UK.
[19]Beatson West of Scotland Cancer Centre, Glasgow, UK.
[20]University College Hospital London, London, UK.
[21]Guy's and Saint Thomas' NHS Foundation Trust, London, UK.
*Corresponding authors

[#]Equal contribution

Corresponding Authors:

Claire Harrison. Guy's and St Thomas' NHS Foundation Trust, Westminster Bridge Road, London, SE1 7EH, UK. Phone: +44(0)207 1882742. Fax: +44(0)207 1882728. E-mail: Claire.Harrison@gstt.nhs.uk

Simon Mendez-Ferrer. University of Cambridge and NHS Blood and Transplant, Wellcome-MRC Cambridge Stem Cell Institute, Jeffrey Cheah Biomedical Centre, Puddicombe Way, Cambridge CB2 0AW, UK. Phone: +44(0)1223 766985. E-mail: sm2116@cam.ac.uk

Supplementary Figure 1

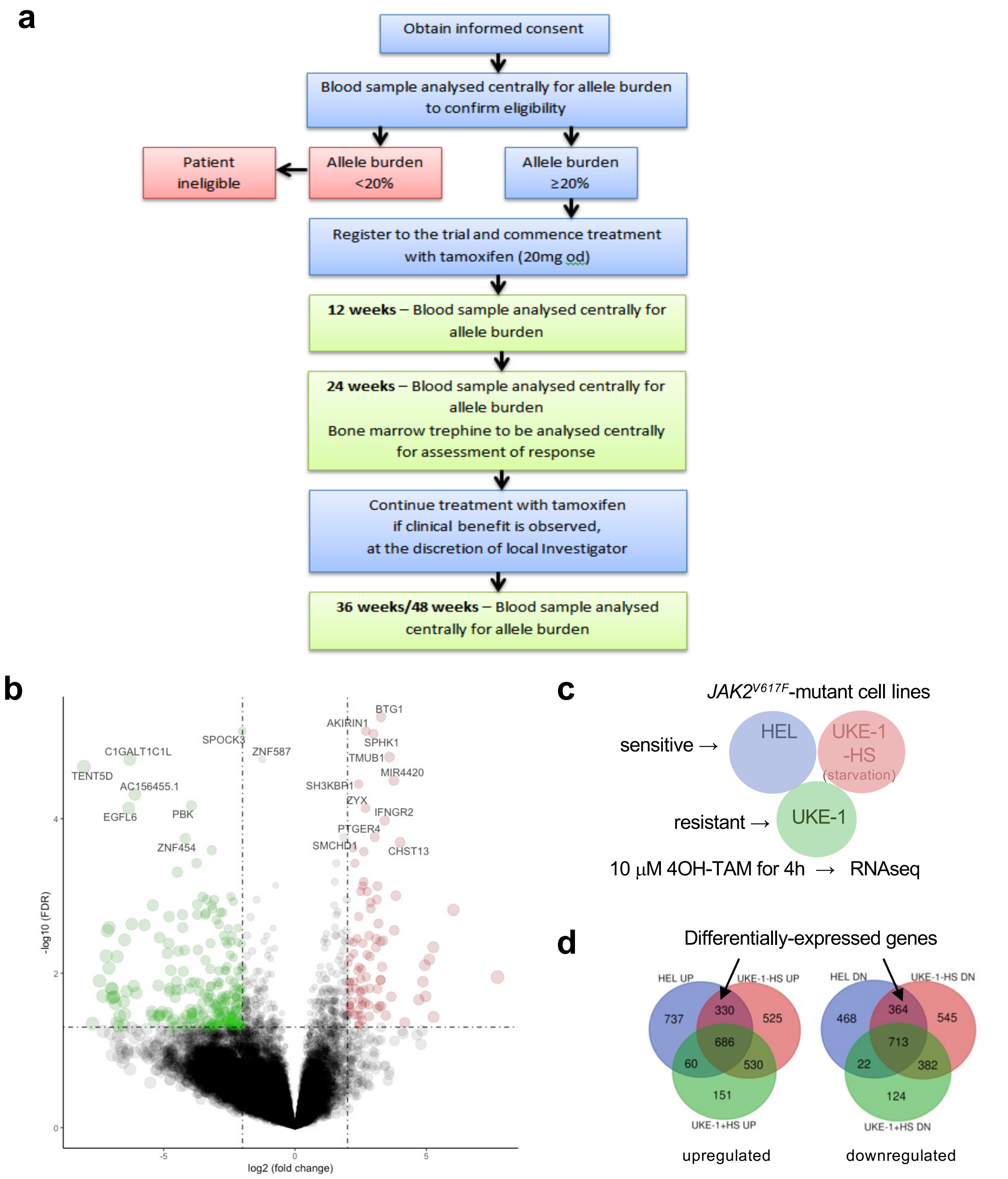

Supplementary Figure 1. Trial scheme and differentially-expressed genes between responders and non-responders.

a Trial scheme for eligibility and central analysis.

b Volcano plot of differentially-expressed genes in HSPCs from responders (red) and non-responders (green) at baseline. The top 19 genes are listed.

c RNA-Seq was performed in tamoxifen-sensitive (HEL and starved UKE-1) and tamoxifen-resistant (UKE-1) cells 4h after treatment with 10 μM 4OH-TAM (n = 3 independent experiments).

d Venn diagram showing differentiated-expressed genes (FDR<0.01; vehicle vs. 4OH-TAM).

Supplementary Figure 2

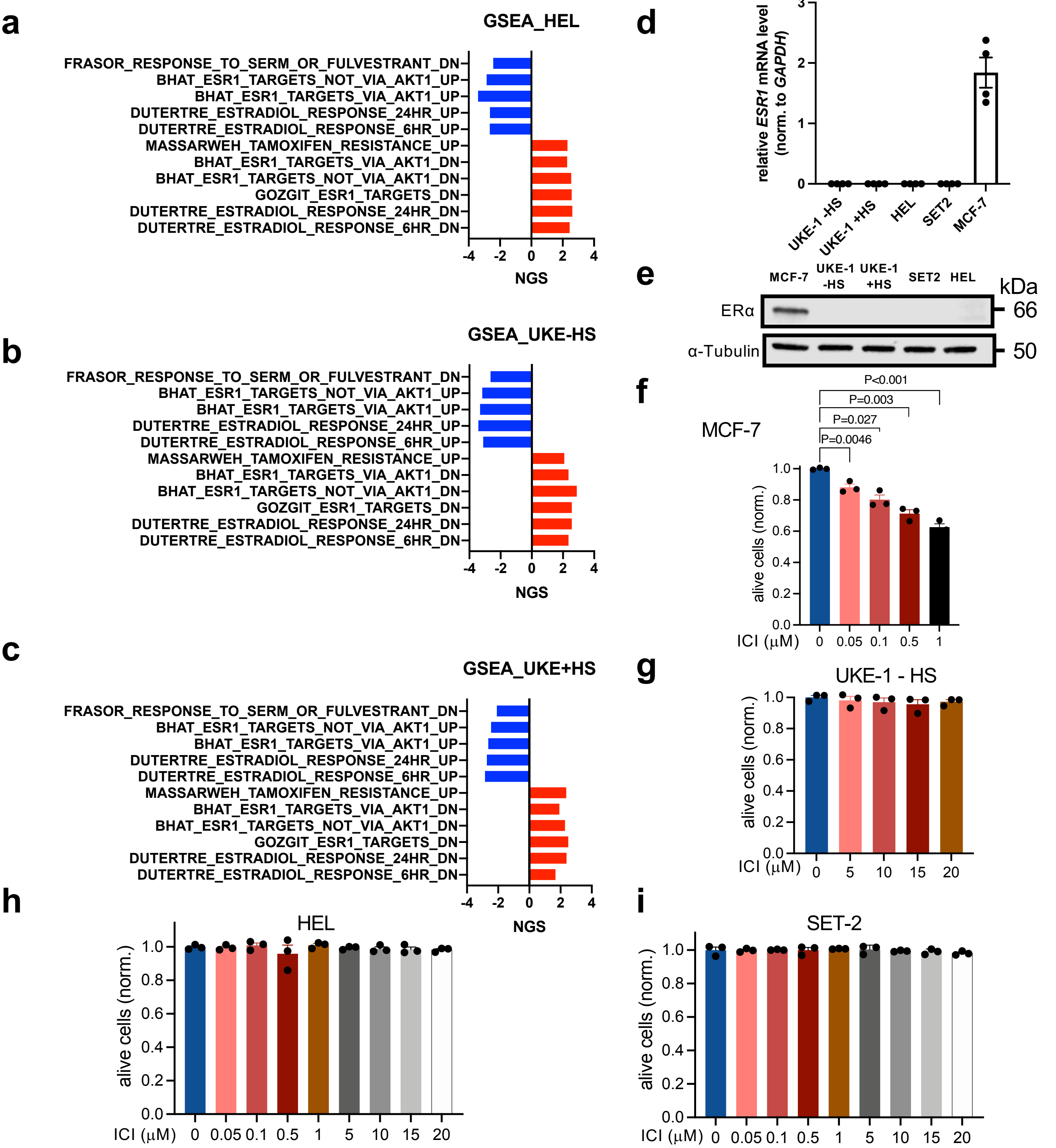

Supplementary Figure 2. ERα does not explain the proapoptotic effect of tamoxifen in MPN.
a-c Gene set enrichment analysis (GSEA) of (**a**) HEL cells, (**b**) horse serum (HS) starved UKE-1 cells and (**c**) UKE-1 cells cultured with competent medium (+HS) treated with 4OH-TAM for 4h suggests the inhibition of genes positively regulated by estradiol or ERα (encoded by *ESR1*), and increased expression of genes downregulated by estradiol or ERα (qvalues<0.005; vehicle vs 4OH-TAM) (*n* = 4 independent experiments).
d-e ERα is not expressed in *JAK2*^{V617F}-mutated human cell lines. *ESR1* mRNA (**d**) and ERα protein (**e**) were not detectable in UKE-1 cells, HEL cells or SET-2 cells (*n* = 4 independent experiments). MCF-7 breast cancer cells were used as a positive control and α-Tubulin was measured as housekeeping control for Western-Blot.
f-i Treatment with the ERα degrader fulvestrant (ICI 182780) over 24h dose-dependently induces apoptosis of MCF-7 (**f**) breast cancer cells but spares 4OH-TAM-sensitive MPN cells, such as HS-depleted UKE-1 cells (g), HEL cells (**h**) and SET-2 cells (**i**) (*n* = 3 independent experiments).
d,**f-i** Data are mean+SEM; **p<0.01; ***p<0.001; One-way ANOVA and Tukey's test.

Supplementary Figure 3

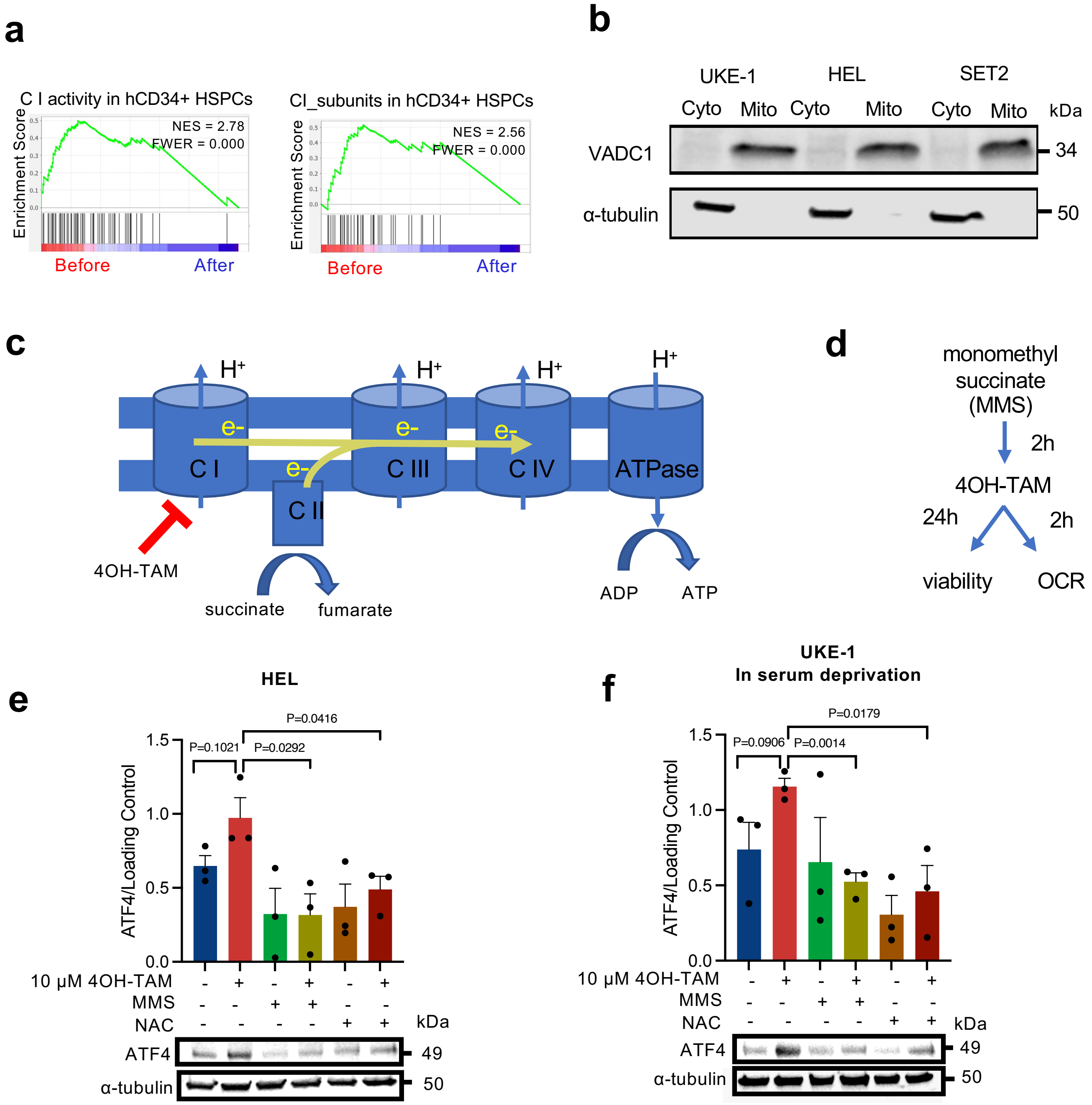

Supplementary Figure 3. Tamoxifen treatment interferes mitochondrial activity and integrated stress response.

a Downregulation of mitochondrial CI-related genes in HSPCs from responders 24w after tamoxifen treatment (*n* = 6 samples). NES, normalized enrichment score. FWER, family-wise error rate.

b Western blot of isolated mitochondria from UKE-1, showing representative markers for cytosolic and mitochondrial fraction. VDAC1, mitochondrial markers, and α-tubulin, cytosolic marker.

c Scheme depicting mitochondrial electron (e-) transport chain and proton (H^+) pumping allowing ATP synthesis; complex (C) I is inhibited by 4OH-TAM, but succinate prodrugs can bypass C I inhibition and restore e- transport and ATP synthesis downstream of C II.

d Experimental paradigm to rescue 4OH-TAM inhibition of OCR or cell viability by monomethyl succinate (MMS).

e-f Quantification (top) and representative Western blots (bottom) of ATF4 and α-tubulin (loading control) before/after 4-hour 10 μM 4OH-TAM treatment in absence or presence of N-acetyl cysteine (NAC) and monomethyl succinate (MMS) in HEL (**e**) and UKE-1 cells cultured without horse serum (**f**) (*n* = 3 independent experiments). Data are mean ± SEM. *p<0.05, **p<0.01, ***p<0.001, ****p<0.0001; One-way ANOVA and Dunnett's test.

Supplementary Figure 4

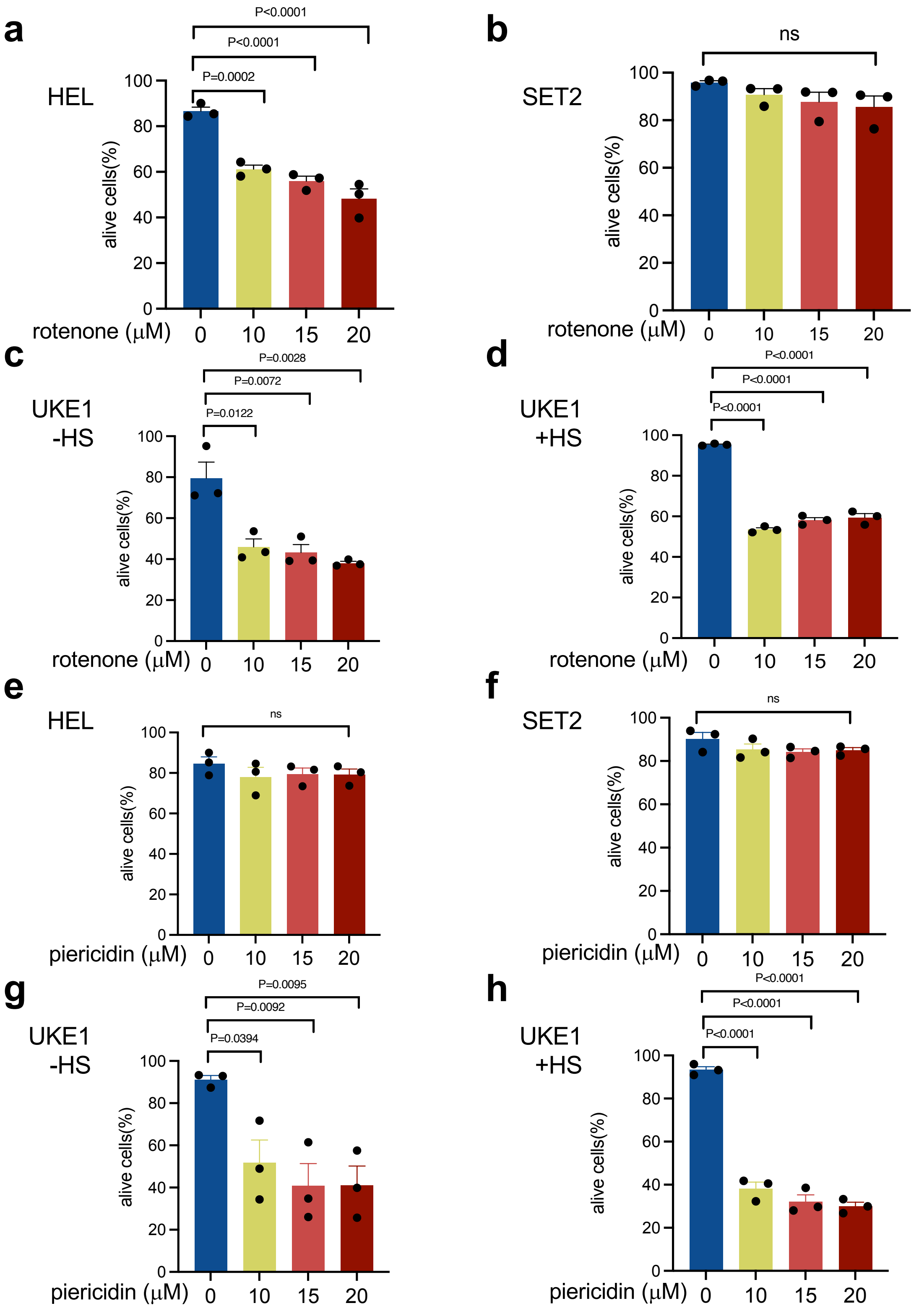

Supplementary Figure 4. Enhanced proapoptotic effect of 4OH-TAM, compared with mitochondrial complex I inhibitors, in human *JAK2*^{V617F}-mutated cell lines.
a-d Treatment with rotenone over 24h induces apoptosis only in HEL cells (**a**), horse-serum-starved (-HS) UKE-1 cells (**c**) and UKE-1 cells in competent medium (**d**), but not in SET2 cells (**b**) (*n* = 3 independent experiments).
e-h Treatment with Piericidin A over 24h induces apoptosis only in horse-serum-starved (-HS) UKE-1 cells (**g**) and UKE-1 cells in competent medium (**h**), but not in HEL cells (**e**) or SET2 cells (**f**) (*n* = 3 independent experiments). Data are mean±SEM; *p<0.05, **p<0.01, ***p<0.001, One-way ANOVA and Tukey's test.

Supplementary Figure 5

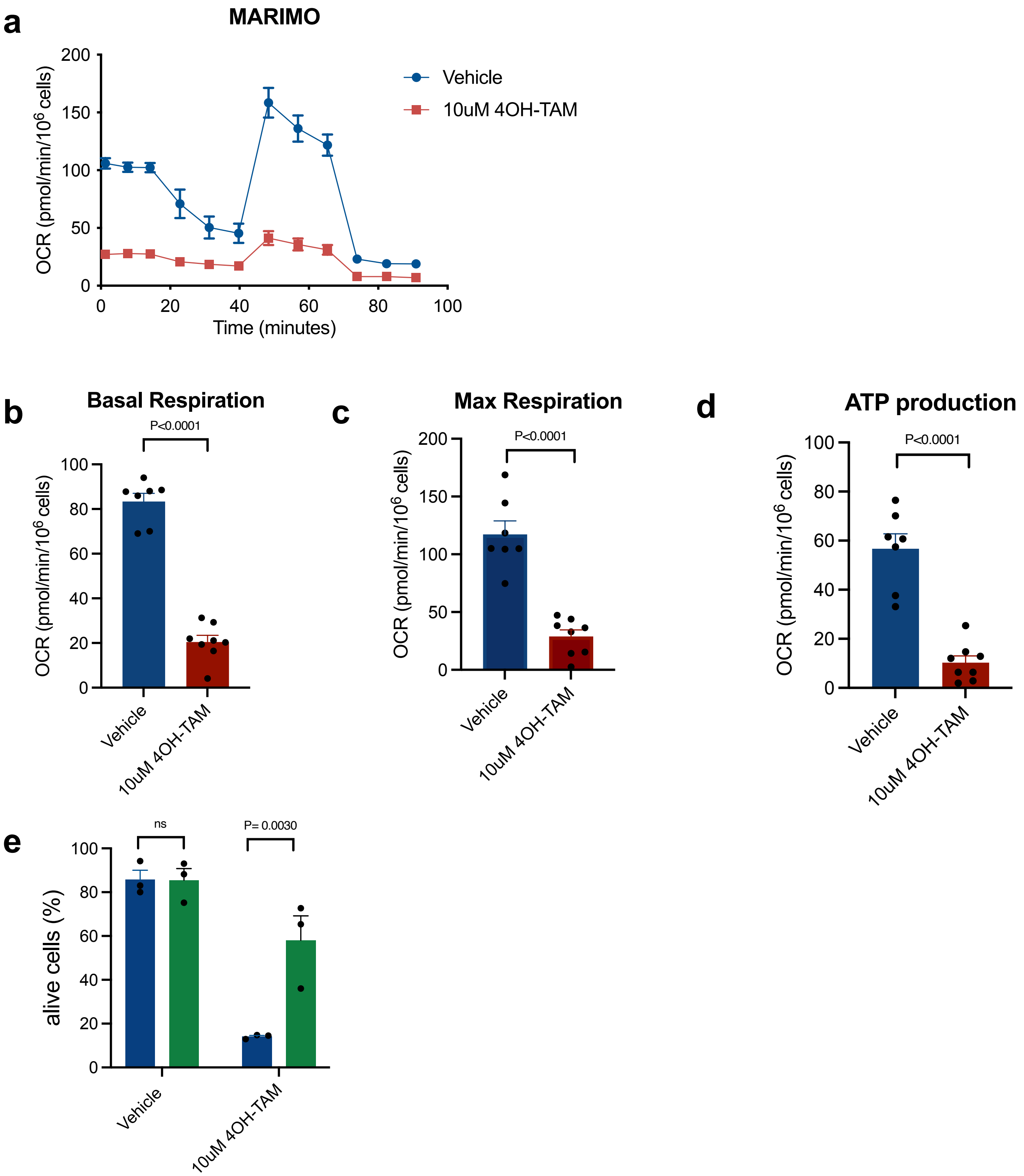

Supplementary Figure 5. Tamoxifen inhibits mitochondrial respiration of MARIMO cells.
a-d 4OH-TAM suppresses the respiration of MARIMO cells, leading to reduced oxygen consumption rate (OCR) (**a**), basal respiration (**b**), maximal respiration (**c**) and OXPHOS-derived ATP generation (**d**). (*n* = 7 independent experiments in vehicle control; *n* = 8 independent experiments). Data are mean ± SEM; Unpaired t-test.
e The activation of respiratory complex II by water soluble succinate (MMS, mono-methyl succinate) bypasses respiratory complex I inhibition and protects cell viability after 4OH-TAM treatment. These results indicate that tamoxifen inhibits respiratory complex I and induces apoptosis in CALR-mutated MARIMO cells similarly to $JAK2^{V617F+}$ cells. *n* = 3 independent experiments. Data are mean ± SEM, Two-way ANOVA and Dunnett's test.

Supplementary Note

TAMARIN

Effects of TAMoxifen on the Mutant Allele Burden and Disease Course in Patients with MyeloprolifeRatIve Neoplasms

**Chief Investigator:**	**Professor Claire Harrison**
**Sponsor:**	**University of Birmingham**
**Version 4.0**	**29-Oct-2018**
**EudraCT:**	**2015-005497-38**
**Sponsor's number:**	**RG_15-234**
**CAS Code:**	**HM2056**
**ISRCTN65011803**	

This application is supported by the facilities funded through Birmingham Science City: Translational Medicine Clinical Research Infrastructure and Trials Platform, an Advantage West Midlands (AWM) funded project which forms part of the Science City University of Warwick and University of Birmingham Research Alliance

TRIAL PERSONNEL

Sponsor
University of Birmingham
Correspondence to:
Cancer Research UK Clinical Trials Unit (CRCTU), Centre for Clinical Haematology,
Queen Elizabeth Hospital, Edgbaston, Birmingham B15 2TH
Tel: 0121 371 7866
Fax: 0121 371 7874
Email: TAMARIN@trials.bham.ac.uk

Chief Investigator
Professor Claire Harrison
Consultant Haematologist and Deputy Clinical Director Cancer and Haematology
Guys and St. Thomas' Hospital
London
SE1 9RT
Tel: 0207 188 2742
Fax: 0207 188 2728
Email: Claire.Harrison@gstt.nhs.uk

Co-Investigators

Dr Simon Mendez-Ferrer
Reader in Transfusion Medicine and Group Leader
Department of Haematology
University of Cambridge
Cambridge Biomedical Campus
Cambridge
CB2 0PT
Tel: 01223 588 113
Email: sm2116@medschl.cam.ac.uk

Dr Anna Godfrey
Consultant Haematologist
Department of Haematology
Cambridge University Hospitals
Hills Road
Cambridge
CB2 0QQ
Tel: 01223 217 073
Email: anna.godfrey1@nhs.net

Co-Investigator and Senior Statistician
Aimee Houlton
Cancer Research UK Clinical Trials Unit
Institute of Cancer and Genomic Sciences
University of Birmingham
Birmingham, B15 2TT
Tel: 0121 414 8328
Fax: 0121 414 6061
Email: a.e.houlton@bham.ac.uk

Haematology TAP Team Leader
Sonia Fox
Cancer Research UK Clinical Trials Unit
Centre for Clinical Haematology
Queen Elizabeth Hospital
Edgbaston
Birmingham, B15 2TH
Tel: 0121 371 4366
Fax: 0121 371 7874
Email: s.fox.2@bham.ac.uk

Senior Trial Coordinator
Rebecca Bishop
Cancer Research UK Clinical Trials Unit
Centre for Clinical Haematology
Queen Elizabeth Hospital
Edgbaston
Birmingham, B15 2TH
Tel: 0121 371 7868
Fax: 0121 371 7874
Email: TAMARIN@trials.bham.ac.uk

Central Laboratory
Dr E Joanna Baxter
Lead Scientist/Senior Research Associate
Cambridge Blood and Stem Cell Biobank
University of Cambridge
Department of Haematology
NHS-BT Cambridge Centre
Long Road
Cambridge, CB2 0PT
Tel: 01223 588048
Email: ejb60@medschl.cam.ac.uk

Trial Coordinator
Mirjana Sirovica
Cancer Research UK Clinical Trials Unit
Centre for Clinical Haematology
Queen Elizabeth Hospital
Edgbaston
Birmingham, B15 2TH
Tel: 0121 371 7866
Fax: 0121 371 7874
Email: TAMARIN@trials.bham.ac.uk

Trial Statistician
Rebecca Boucher
Cancer Research UK Clinical Trials Unit
Institute of Cancer and Genomic Sciences
University of Birmingham
Birmingham, B15 2TT
Tel:0121 414 9065
Fax: 0121 414 6061
Email: R.H.Boucher@bham.ac.uk

Clinical Coordinators
Professor Claire Harrison and Dr Anna Godfrey

SIGNATURE PAGE

TAMARIN Trial Protocol v4.0 29-Oct-2018

This protocol has been approved by:

Name: Professor Claire Harrison **Trial Role:** Chief Investigator

Signature: 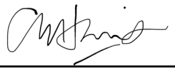 **Date:** 11/29/2018 9:08:51 AM EST DD / MON / YYYY

This protocol describes the TAMARIN trial and provides information about procedures for patients taking part in the TAMARIN trial. The protocol should not be used as a guide for treatment of patients not taking part in the TAMARIN trial.

AMENDMENTS

The following amendments and/or administrative changes have been made to this protocol since the implementation of the first approved version

Amendment number	Date of amendment	Protocol version number	Type of amendment	Summary of amendment
3	28-Apr-2017	2.0	Substantial	Clarification of eligibility criteria<br>Update to outcome measures<br>Update to dose modification rules<br>Clarification of response assessment<br>Inclusion of guidance for treatment continuation/discontinuation
5	08-Mar-2018	3.0	Substantial	Extension of recruitment period and trial duration<br>Update to outcome measures<br>Update to schedule of assessments<br>Update of guidance for treatment continuation<br>Clarification of exploratory outcomes
N/A	15-Aug-2018	3.0a	Notification	Change in Data Protection Regulations
7	29-Oct-2018	4.0	Substantial	Update to secondary outcomes, update to schedule of events and assessments

TRIAL SYNOPSIS

Title

Effects of Tamoxifen on the Mutant Allele Burden and Disease Course in Patients with Myeloproliferative Neoplasms (MPN)

Trial Design

A multicentre phase II, single stage A'herns design clinical trial to assess the safety and activity of tamoxifen in MPN.

Objectives

To investigate the activity of tamoxifen in reducing the molecular markers of disease burden in MPN. The trial will also look at the safety of tamoxifen in this patient population.

Outcome Measures

Primary outcome

- Reduction in the peripheral blood JAK2-V617F, CALR 5bp insertion (exon 9), or CALR 52bp deletion (exon 9) mutant allele burden of ≥50% at 24 weeks

Secondary outcomes

- Reduction in the peripheral blood mutant allele burden of ≥50% at 12 weeks
- Toxicity measured as the number of grade 3 and 4 adverse events reported
- Thrombotic events of any grade reported and validated

- Haematological response at weeks 12 and 24 [1-2]
- Proportion of patients in each response category according to IWG-MRT response criteria for MF [2] and 2013 ELN response criteria for ET/PV [3] at 24 weeks
- Proportion of patients showing an improvement in response category at 24 weeks compared to baseline [1-2]

Exploratory outcomes

- Change in allele burden between weeks 12, 24 and baseline.
- Proportion of patients showing a decrease in requirement for cytoreduction at 24 weeks compared to baseline
- Proportion of patients showing a decrease in allele burden of ≥50% at 36 and 48 weeks compared to baseline
- Duration of reduction in the mutant allele burden, defined as time from first observed reduction of ≥50% until reduction from baseline becomes <25% or patients death.
- Expression (RNAseq), DNA-protein interaction (ChipSeq) and methylation studies focused on oestrogen receptor signalling in haematopoietic progenitors obtained from peripheral blood and bone marrow before (peripheral blood only) and after tamoxifen treatment

Patient Population

Patients with an MPN with at least a 20% JAK2-V617F, CALR 5bp insertion (exon 9) or CALR 52bp deletion (exon 9) allele burden in peripheral blood granulocyte DNA (as determined centrally), stable disease and meet the eligibility criteria.

Sample Size

A total of 42 patients will be recruited over 28 months. Of the 42 patients, we will aim to achieve a minimum of 15 PV patients, 10 ET patients and 5 MF patients.

Main Inclusion and Exclusion Criteria

Inclusion Criteria

- Age ≥ 60 years (men aged between 50-59 may also be considered following discussion with the Chief Investigator)
- Women must be post-menopausal (defined as amenorrhoeic for at least 12 consecutive months following cessation of all exogenous hormonal treatments)
- Confirmed diagnosis of JAK2-V617F, CALR 5bp insertion (exon 9), or CALR 52bp deletion (exon 9) positive Essential Thrombocythaemia (ET), Polycythaemia Vera (PV) or Myelofibrosis (MF) (primary or secondary) for ≥ 6 months
- JAK2-V617F, CALR 5bp insertion (exon 9), or CALR 52bp deletion (exon 9) mutant allele burden ≥ 20% in peripheral blood granulocyte DNA at study entry (**assessed via central review**)
- WHO performance status 0-2 (see Appendix 1)
- For patients with PV or ET, maintenance of platelet count ≤600 x 10^9/L, WBC ≤25 x 10^9/L and venesection requirements ≤1 per month for the previous 3 months prior to registration, without introduction of any new therapeutic agents for their MPN for 6 months prior to registration.
- For patients with MF, there must not have been any evidence of disease progression* for the previous 6 months (prior to registration) and no new therapeutic agents for their MPN introduced during this period
- Patients receiving cytoreductive therapy (with the exception of interferon alpha or investigational agents) for their MPN (not solely aspirin or venesection)
- Adequate hepatic function, defined as:
  - bilirubin ≤ 1.5 x upper limit of normal (ULN) (patients with elevated bilirubin due to Gilbert's syndrome are eligible)
  - AST/ALT/ALP ≤ 2.5 x ULN
- Adequate renal function (creatinine clearance >30 mL/min)

- Male patients must agree to use effective contraception during participation in the trial and for 2 months after the last dose of trial treatment
- Patient must be able to give written informed consent

*Defined by IWG-MRT ELN criteria (Appendix 6). Please note no baseline bone marrow is required to confirm absence of “Leukemic transformation confirmed by a bone marrow blast count of ≥20%”.

Exclusion Criteria

- Leukaemic transformation (>20% blasts in blood, marrow or extramedullary site)
- Accelerated phase of disease as indicated by ≥10% blasts in the peripheral blood
- Treatment of ET, PV or MF with Interferon alpha or other investigational agents for their MPN within 6 months prior to trial entry. JAK inhibitors, such as ruxolitinib, are allowed if taken continuously for ≥6 months prior to registration (dose changes during that period will be allowed)
- Any of the following previous thrombotic events at any time:
    - Portal or other splanchnic venous thrombosis
    - Vascular access complication
    - Ischemia cerebrovascular
    - Stroke
    - Transient Ischaemic attack
    - Superficial thrombophlebitis
    - Venous Thromboembolic events including pulmonary embolism (PE) and deep vein thrombosis (DVT)
    - Peripheral vascular ischemia
    - Visceral arterial ischemia
    - Acute coronary syndrome
    - Myocardial infarction
- Previous malignancy within 5 years with the exception of adequately treated cervical carcinoma in situ or localized non-melanoma skin cancer
- Previous endometrial cancer, hyperplasia or polyps
- Prior treatment with hematopoietic stem cell transplantation
- Patients who do not carry JAK2-V617F,CALR 5bp insertion (exon 9) or CALR 52bp deletion (exon 9) mutations or whose allele burden is <20% at study entry (**assessed via central review**)
- Female patients receiving hormone replacement therapy
- Hypertriglyceridemia > grade 1
- Any serious underlying medical condition (at the judgment of the Investigator), which could impair the ability of the patient to participate in the trial (e.g. liver disease, active autoimmune disease, uncontrolled diabetes, uncontrolled infection (HIV, Hepatitis B and C), known genetic defect (apart from MPN) relating to venous thromboembolic events, or psychiatric disorder precluding understanding of trial information)
- Known hypersensitivity to tamoxifen or hypersensitivity to any other component of tamoxifen
- Concomitant drugs contraindicated for use with the trial drug according to the Summary of Product Characteristics (Appendix 8)
- Known planned scheduled elective surgery during study with the exception of dental and low risk eye surgery (e.g. cataracts)

Trial Duration

All patients will receive trial treatment for 24 weeks. Following 24 weeks, they will be able to continue treatment with tamoxifen if there is clinical benefit, at the discretion of the treating Investigator, and their response will be reassessed after 36 and 48 weeks of treatment as applicable. The last patient visit will be 28 days after the last administration of trial treatment.

Recruitment will be over 28 months from 13-15 trial centres. Therefore the total trial duration will be 35 months for patients receiving treatment for 24 weeks. For patients continuing treatment for 36 and 48 weeks, the total trial duration will be 38 and 41 months, respectively.

Trials Office Contact Details

TAMARIN Trial Office
CRCTU, Centre for Clinical Haematology,
Queen Elizabeth Hospital, Edgbaston, Birmingham, B15 2TH
Tel: 0121 371 7866 Fax: 0121 371 7874 Email: TAMARIN@trials.bham.ac.uk

Trial Schema for Eligibility and Central Analysis

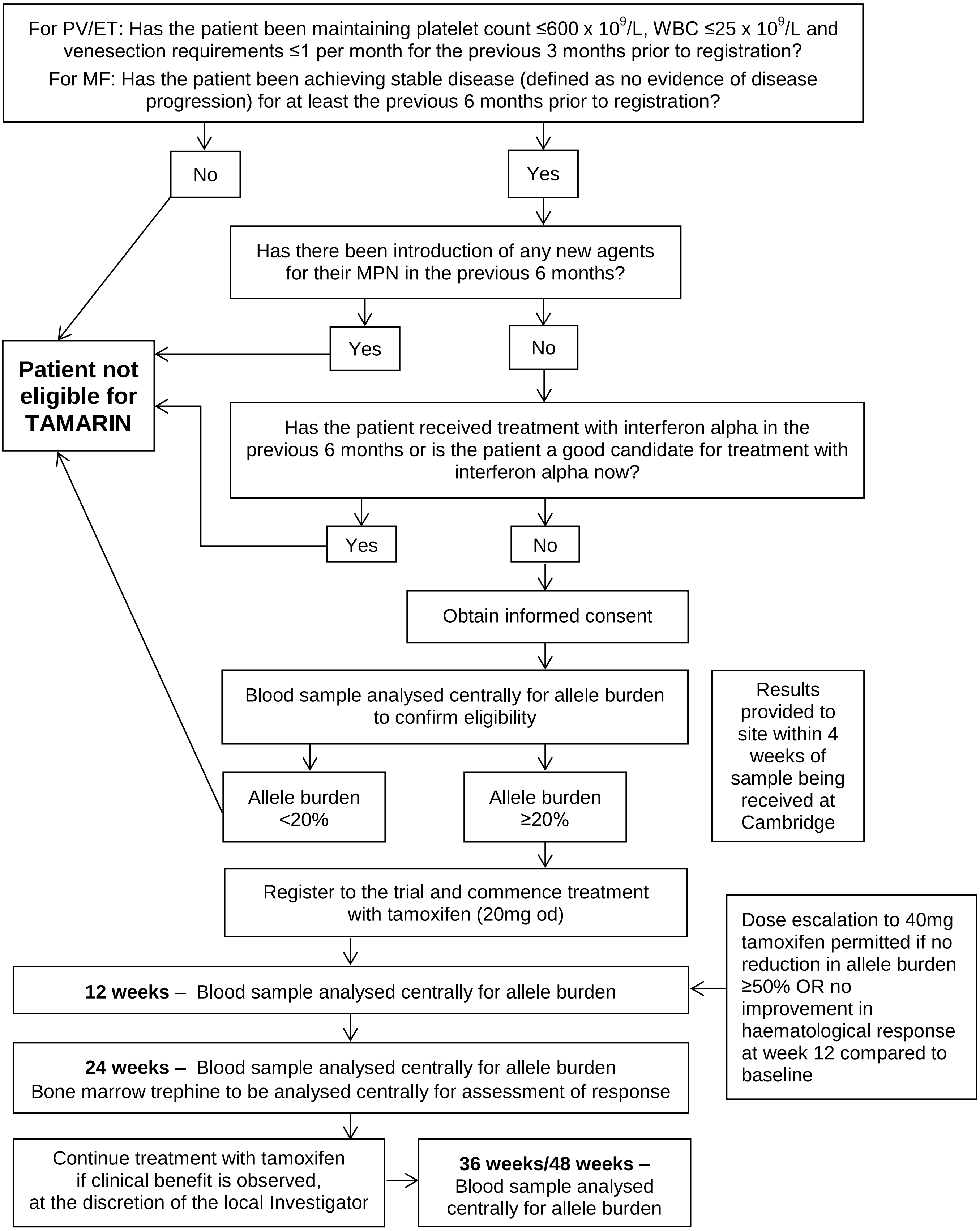

Schedule of Events	Baseline (within 6 weeks of registration)	4 weeks[9]	12 weeks	18 weeks[9, 10]	24 weeks	Treatment beyond 24 weeks	End of study visit
						Follow-up minimum of 12 weekly	28 days after last treatment[5]
Informed consent	x[1]						
Demographic data and medical history[3] including prior diagnosis/prior treatment.	X						
Medical examination (including height (baseline only), weight, blood pressure, WHO performance status, palpation of liver and spleen)	X	X	X	X	X	X	X
Ultrasound to assess spleen	X				X[4]		
Haematology and Biochemistry[2]	X	X	X	X	X	X	X
Assessment of Haematological response[6]	X	X	X	X	X	X	X
Full fasting lipid profile including triglycerides	X		X		X		
MPN-SAF TSS (QoL)	X				X		
Administration of tamoxifen		<<Continuous 20mg once daily from registration>>[7] *Consider increasing to 40mg once daily at week 18 if appropriate*				If clinically indicated	
Blood samples for central review of allele burden	X		X		X	X[8]	
Bone marrow aspirate and trephine sample collection					X		
Record of venesections (for PV) or transfusions (for MF) received		<<Continuous throughout study>>					
Concomitant diseases and treatment		<<Continuous throughout study>>					
Adverse events		<<Continuous throughout study>>					

[1] Can be obtained more than 6 weeks prior to registration. [2] To include Hb, HCT, WBC count, RBC count , Platelets, ANC, lymphocytes, blood film, eGFR/calculated creatinine clearance, ALP, AST/ALT, bilirubin, calcium, LDH. [3] Particular attention must be paid to gynaecological history. [4] For PV/ET patients that entered the trial with PR and enlarged spleen, the ultrasound at 24 weeks will be performed earlier if a CR is suspected. [5] This applies to 28 days after 24 weeks of treatment or 28 days after the last treatment if patient receives treatment for more/for less than 24 weeks. [6] Please refer to section 7.2.9. [7] Please refer to section 7.4 for dose modification rules. [8] For patients continuing treatment beyond 24 weeks, mutant allele burden will be reassessed at 36 weeks. For patients continuing treatment beyond 36 weeks, mutant allele burden will be reassessed at 48 weeks. Please refer to sections 7.3.2 and 7.8 for further details. [9] Every effort should be made for

patients to attend on the scheduled visit days. However, if a patient is unable to attend on the specified day, assessments may be scheduled for +/- 7 days. [10] This visit may be performed via telephone should this be more convenient for the patient and a medical examination and haematology and biochemistry assessments will not be required. However, the patient should attend this visit should there be a need for a dose escalation or de-escalation as this should be reviewed and implemented at this visit. Patient should also be asked for any symptoms, AEs, SAEs, thrombotic events, etc.

ABBREVIATIONS

AE	ADVERSE EVENT
ALP	ALKALINE PHOSPHATASE
ALT	ALANINE TRANSAMINASE
ANC	ABSOLUTE NEUTROPHIL COUNT
AR	ADVERSE REACTION
AST	ASPARTATE TRANSAMINASE
CALR	CALRETICULIN
CBSB	CAMBRIDGE BLOOD AND STEM CELL BIOBANK
CR	COMPLETE RESPONSE
CRF	CASE REPORT FORM
CR UK	CANCER RESEARCH UK
CRCTU	CANCER RESEARCH UK CLINICAL TRIALS UNIT (UNIVERSITY OF BIRMINGHAM)
CTCAE	COMMON TERMINOLOGY CRITERIA FOR ADVERSE EVENTS
CV	CURRICULUM VITAE
DCF	DATA CLARIFATION FORM
DNA	DEOXYRIBOSE NUCLEIC ACID
DSUR	DEVELOPMENT SAFETY UPDATE REPORT
DVT	DEEP VEIN THROMBOSIS
ECOG	EASTERN COOPERATIVE ONCOLOGY GROUP
eCRF	ELECTRONIC CASE REPORT FORM
eGFR	ESTIMATED GLOMERULAR FILTRATION RATE
eRDC	ELECTRONIC REMOTE DATA CAPTURE
ET	ESSENTIAL THROMBOCYTHAEMIA
FBC	FULL BLOOD COUNT
GCP	GOOD CLINICAL PRACTICE
GP	GENERAL PRACTITIONER
HB	HAEMOGLOBIN
HC	HYDROXYCARBAMIDE
HCT	HAEMATOCRIT
ICF	INFORMED CONSENT FORM
ISF	INVESTIGATOR SITE FILE
IMP	INVESTIGATIONAL MEDICINAL PRODUCT
ISRCTN	INTERNATIONAL STANDARD RANDOMISED CLINICAL TRIAL NUMBER
IWG	INTERNATIONAL WORKING GROUP
IWG-MRT	INTERNATIONAL WORKING GROUP – MYELOPROLIFERATIVE NEOPLASMS RESEARCH AND TREATMENT
JAK	JANUS KINASE
LDH	LACTATE DEHYDROGENASE
MF	MYELOFIBROSIS
ML	MILLILITRE

MHRA	MEDICINES AND HEALTHCARE PRODUCTS REGULATORY AGENCY
MPL	MYELOPROLIFERATIVE LEUKAEMIA
MPN	MYELOPROLIFERATIVE NEOPLASMS
NIHR	NATIONAL INSTITUTE FOR HEALTH RESEARCH
OD	ONCE DAILY
PE	PULMONARY EMBOLISM
PI	PRINCIPAL INVESTIGATOR
PIS	PATIENT INFORMATION SHEET
PMF	PRIMARY MYELOFIBROSIS
PO	BY MOUTH (PER OS)
PR	PARTIAL RESPONSE
PV	POLYCYTHAEMIA VERA
QoL	QUALITY OF LIFE
R&D	RESEARCH AND DEVELOPMENT
RNA	RIBOSE NUCLEIC ACID
RBC	RED BLOOD CELL
REC	RESEARCH ETHICS COMMITTEE
SAE	SERIOUS ADVERSE EVENT
SAF	SYMPTOM ASSESSMENT FORM
SAP	STATISTICAL ANALYSIS PLAN
SAR	SERIOUS ADVERSE REACTION
SPC	SUMMARY OF PRODUCT CHARACTERISTICS
SUSAR	SUSPECTED UNEXPECTED SERIOUS ADVERSE REACTION
TAP	TRIALS ACCELERATION PROGRAMME
TET2	TET METHYLCYTOSINE DIOXYGENASE 2
TMG	TRIAL MANAGEMENT GROUP
TSC	TRIAL STEERING COMMITTEE
ULN	UPPER LIMIT OF NORMAL
WBC	WHITE BLOOD CELL
WHO	WORLD HEALTH ORGANIZATION
WMA	WORLD MEDICAL ASSEMBLY

Table of contents

TRIAL PERSONNEL **2**
SIGNATURE PAGE **4**
AMENDMENTS **5**
Trial Synopsis **5**
Title 5
Trial Design 5
Objectives 5
Outcome Measures 5
Primary outcome 5
Secondary outcomes 5
Exploratory outcomes 6
Patient Population 6
Sample Size 6
Main Inclusion and Exclusion Criteria 6
Inclusion Criteria 6
Exclusion Criteria 7
Trial Duration 7
Trials Office Contact Details 8
Trial Schema for Eligibility and Central Analysis 9
Schedule of Events 10
Abbreviations **12**
1. Background and Rationale **17**
1.1 Background 17
1.2 Trial Rationale 18
1.2.1 Justification for patient population 18
1.2.2 Justification for design 18
1.2.3 Choice of treatment 18
2. Aims, Objectives and Outcome Measures **19**
2.1 Aims and Objectives 19
2.2 Outcome Measures 19
Primary outcome 19
Secondary outcomes 19
2.2.1 Exploratory outcome measures 19
3. Trial Design **19**
4. Eligibility **20**
4.1 Inclusion Criteria 20
4.2 Exclusion Criteria 20
5. Screening and Consent **21**
5.1 Screening 21
5.1.1 Screening Assessments 21
5.2 Informed Consent 22
6. Trial Entry **22**
7. Treatment Details **23**
7.1 Trial Treatment 23
7.2 Assessments 23
7.2.1 Demographic data and medical history 23
7.2.2 Medical examination/symptom assessment 23

7.2.3 Ultrasound ... 23
7.2.4 Haematology ... 24
7.2.5 Biochemistry ... 24
7.2.6 Lipid profile ... 24
7.2.7 Bone marrow evaluation ... 24
7.2.8 Quality of Life ... 24
7.2.9 Assessment of response ... 24
7.3 Sample Collection ... 25
7.3.1 Bone marrow samples ... 25
7.3.2 Blood samples ... 25
7.4 Dose Modifications ... 25
7.5 Treatment Compliance ... 26
7.6 Supportive Treatment ... 26
7.7 Concomitant Medication ... 26
7.8 Patient Follow Up ... 26
7.9 Patient Withdrawal ... 26
8. Adverse Event Reporting ... 27
8.1 Reporting Requirements ... 27
8.1.1 Adverse Events ... 27
8.1.2 Serious Adverse Events ... 28
8.1.3 Reporting period ... 28
8.2 Reporting Procedure ... 28
8.2.1 Site ... 28
8.2.2 Trials Office ... 29
8.2.3 Reporting to the Competent Authority and main Research Ethics Committee ... 29
8.2.4 Investigators ... 29
8.2.5 Trial Steering Committee ... 29
9. Data Handling and Record Keeping ... 29
9.1 Data Collection ... 29
9.2 Archiving ... 31
10. Quality Management ... 31
10.1 Site Set-up and Initiation ... 31
10.2 On-site Monitoring ... 31
10.3 Central Monitoring ... 31
10.4 Audit and Inspection ... 31
10.5 Notification of Serious Breaches ... 31
11. End of Trial Definition ... 32
12. Statistical Considerations ... 32
12.1 Definition of Outcome Measures ... 32
12.1.1 Primary outcome measure ... 32
12.1.2 Secondary outcome measures ... 32
12.1.3 Exploratory outcome measure ... 32
12.2 Analysis of Outcome Measures ... 33
12.2.1 Primary outcome measure ... 33
12.2.2 Secondary outcome measures ... 33
12.2.3 Exploratory outcome measure ... 33
12.3 Planned Sub Group Analyses ... 34
12.4 Planned Interim Analysis ... 34

12.5 Planned Final Analyses ... 34
12.6 Power Calculations ... 34
13. Trial Organisational Structure ... 34
13.1 Sponsor ... 34
13.2 Coordinating Centre ... 34
13.3 Trial Management Group ... 34
13.4 Trial Steering Committee ... 35
13.5 Finance ... 35
14. Ethical Considerations ... 35
15. Confidentiality and Data Protection ... 35
16. Insurance and Indemnity ... 36
17. Publication Policy ... 36
18. Reference List ... 36
Appendix 1 – WHO Performance Status ... 38
Appendix 2 - WMA Declaration of Helsinki ... 39
Appendix 3 - Definition of Adverse Events ... 42
Appendix 4 - Common Toxicity Criteria Gradings ... 44
Appendix 5 – European LeukaemiaNet Criteria for Response to therapy in PV and ET ... 45
Appendix 6 – Revised Response Criteria for Myelofibrosis ... 46
Appendix 7 – Revised response criteria for polycythemia vera and essentialthrombocythemia: an ELN and IWG-MRT consensus project ... 48
Response criteria for PV ... 48
Response criteria for ET ... 49
Appendix 8 – List of Prohibited Concomitant Medication and Link to Tamoxifen SPC ... 50
Appendix 9 – MPN Symptom Assessment Form Total Symptom Score (MPN-SAF TSS) ... 51

1. BACKGROUND AND RATIONALE

1.1 Background

The myeloproliferative neoplasms (MPN) comprise several clonal haematological malignancies including polycythemia vera (PV), essential thrombocythemia (ET) and primary myelofibrosis (PMF). MPN results from transformation of a haematopoietic progenitor cell and are characterised by overproduction of mature functional blood cells and have a chronic clinical course. Patients are at increased risk of arterial and venous thrombosis, major haemorrhage and, in the longer term, transformation to myelofibrosis and/or acute leukaemia. It is estimated that approximately 3,000 new patients are diagnosed with MPN per annum in the UK [4].

There are currently no curative options for patients with MPN other than stem cell transplant which is only suitable for a minority of patients. Instead our current therapies are mainly aimed at controlling the abnormal numbers of blood cells and reducing the risk of thrombotic and haemorrhagic events without exposing patients to increased risk of transformation into myelofibrosis or leukaemia [5].

Venesection and low dose aspirin are accepted as the standard of care for initial therapy with the addition of hydroxycarbamide (HC) to reduce the platelet count [6]. Current research in this area is mainly focussed upon JAK inhibition and the use of interferon. These trials are only open to patients with newly diagnosed high risk MPN (MPD RC112 interferon vs hydroxycarbamide) or those who have become resistant or refractory to hydroxycarbamide (MAJIC). None of these studies are targeting reduction of the abnormal disease clone. More recently JAK inhibitor therapy has become an option for some patients. Ruxolitinib is licenced for the treatment of disease-related splenomegaly or symptoms in adult patients with PMF or post ET/PV-MF and has also recently been licensed for the treatment of adult patients with PV who are resistant to or intolerant of hydroxycarbamide. However, access is very limited due to cost, especially in patients with PV and this approach does not offer a cure.

In this trial we are therefore seeking to try to build upon the success of current therapies, which reduce risks of thrombosis and haemorrhage, and find a therapy which moves the treatment target towards a molecular eradication of the disease which is very rarely achieved with current therapies.

The discovery of the JAK2-V617F mutation provided the first genetic marker of the malignant clone in MPN. It is present in >95% of those with PV and approximately 50-60% of ET and PMF patients [7, 8]. JAK2 activation is a consistent pathological finding in MPN, even in those patients without the JAK2-V617F mutation. There is good evidence that JAK2 activation drives much of the platelet and leukocyte activation found in these disorders, and is associated with thrombosis, especially venous events [9]. Furthermore, the presence of the JAK2-V617F mutation is associated with poorer survival in patients with PMF [8]. There is also evidence that a high JAK2-V617F mutant allele burden is associated with features of more advanced disease [10, 11] in particular, it is associated with factors such as thrombosis [12] and myelofibrotic transformation [13] which result in poor outcomes for patients.
More recently, it was also discovered that CALR mutations were present in 25% and 35% of patients with ET and PMF respectively [14]. When looking at patients without JAK2 or MPL mutations, the incidence of CALR mutations is as high as 70- 84% [15]. The impact of a high CALR mutant allele burden has not yet been elucidated.

The potential role of tamoxifen in treating MPN has recently been assessed *in vivo* by the Mendez Ferrer group. These data indicate that accumulation of abnormal blood cells and bone marrow degeneration (caused by MPN) are both blocked in mouse models by treatment with tamoxifen. Tamoxifen mimics the effects of oestrogen and is able to regulate the survival, proliferation and self-renewal of stem cells that give rise to blood cancers. In MPN mouse models, tamoxifen treatment blocked the excessive production of blood cells by restoring normal levels of programmed cell death in mutant blood progenitor cells. The effects of tamoxifen were more pronounced in the mutated cells than in the normal cells, allowing for a reduction in the tumour size [16].

Polymorphisms in the oestrogen receptor-alpha have been suggested to confer susceptibility to develop acute myeloid leukaemia. This gene is also frequently methylated in this disease and its methylation status might predict prognosis. Its re-expression after treatment with hypomethylating agents also correlates with clinical response [17-21]. The results of the correlative science will also be potentially important in assessing which patients might benefit from tamoxifen therapy.

1.2 Trial Rationale

1.2.1 Justification for patient population

This trial will be open to all patients with MPN who have a measureable disease burden (defined by quantifiable abnormal clone of ≥20% mutated JAK2-V617F, CALR 5bp insertion (exon 9) or CALR 52bp deletion (exon 9)).

The eligibility criteria have also been carefully considered to take into account the well-established safety profile of tamoxifen and the trial is therefore open only to males and post-menopausal women.

Patients with a prior thrombosis will also be excluded as both tamoxifen and MPN are associated with a higher risk of thrombosis. To try and ensure patients have stable blood counts whilst on trial, and again minimise the risk of thrombosis, patients will also be able to continue with existing therapy for their MPN such as hydroxycarbamide, venesection, and aspirin. However, patients will not be able to continue/receive concomitant treatment with therapies that lower the allele burden such as interferon alpha) as this would prevent accurate assessment of the primary outcome. JAK inhibitors, such as ruxolitinib, will be allowed if taken continuously for ≥6 months prior to registration, as any changes in allele burden are expected to occur early during treatment.

In addition, only patients whose disease is currently well controlled on standard therapies will be eligible which will further minimise the risk of thrombosis.

Another well described side effect of tamoxifen is women experiencing symptoms of premature menopause and uterine changes. This study will therefore only include post-menopausal women.

1.2.2 Justification for design

This will be the first trial of tamoxifen in MPN and it is designed to gather preliminary evidence of activity.

A safe dose of tamoxifen is well-established in other malignancies and *in vitro* data indicates that this dose would be effective in this population of patients. A single arm phase II study was selected in order to determine evidence of activity in MPN to further evaluate in a randomised phase 3 setting. An Ahern's design was selected to evaluate activity with stopping criteria to evaluate toxicity given that tamoxifen has not been used in this disease area and in combination with cytoreductive agents.

If this study shows a response (reduction in allele burden of ≥50%) in ≥ 10% of patients, it will warrant further investigation in a larger, randomised trial.

In this study, clone size (peripheral blood granulocyte JAK2-V617F, CALR 5bp insertion (exon 9) or CALR 52bp deletion (exon 9) mutant allele burden) reduction by 50% of the baseline value after 24 weeks of treatment will be used as a surrogate endpoint for the effect of tamoxifen on MPN. 50% reduction was chosen as this is an accepted endpoint in the MPN community [22].

This trial will also measure gene expression and methylation and a surrogate of oestrogen receptor signalling, and will correlate the results with disease progression and response to treatment. The goal is to be able to stratify patients according to biomarkers of oestrogen receptor signalling and predictors of clinical response.

1.2.3 Choice of treatment

This trial will test tamoxifen; a cheap, widely available drug, with a well-documented safety profile, in a new disease indication. Tamoxifen is a non-steroidal triphenylethylene-based drug which displays a complex spectrum of oestrogen antagonist and oestrogen agonist like pharmaceutical effects in different tissues. Tamoxifen is an established treatment in breast cancer where it acts primarily as an antioestrogen, preventing oestrogen binding to the oestrogen receptor.

Patients will receive 20mg (the most frequently used dose in breast cancer) once daily PO continuously for a minimum of 24 weeks.

This trial will specifically build on the *in vivo* work performed by the Mendez-Ferrer group. In this *in vivo* work, low dose tamoxifen was used and a strong response was observed. Therefore, the standard dose of 20mg will be used.

The trial will exploit the novel regulation of blood progenitors by oestrogens which is not currently being investigated elsewhere in MPN and is also fundamentally different from the current strategies, since the primary target of therapy is not the specific mutation/s of the stem cells. The JAK2/CALR mutated cells will be stimulated with a drug that mimics the effects of the natural hormone on the blood progenitor cells but has a much higher potency.

We will also correlate the patient response to treatment with the expression and methylation of oestrogen receptors. This could lead to novel and easier treatments and increase our understanding of the clinical evolution of leukaemias.

2. AIMS, OBJECTIVES AND OUTCOME MEASURES

2.1 Aims and Objectives

The aim of this trial is to investigate the activity of tamoxifen in reducing the molecular markers of disease burden in MPN. The trial will also look at the safety of tamoxifen in this patient population.

2.2 Outcome Measures

Primary outcome

- Reduction in the peripheral blood JAK2V617F, CALR 5bp insertion (exon 9) or CALR 52bp deletion (exon 9) mutant allele burden of ≥50% at 24 weeks

Secondary outcomes

- Proportion of patients with a reduction in the peripheral blood JAK2-V617F, CALR 5bp insertion (exon 9), or CALR 52bp deletion (exon 9) mutant allele burden of ≥50% at 12 weeks
- Toxicity measured as the number of grade 3 and 4 adverse events reported.
- The number of thrombotic events of any grade reported and validated.
- Haematological response, assessed at weeks 12 and 24, for patients who enter the study in response (CR or PR). Haematological response is defined according to 2009 ELN criteria for ET/PV patients [1] and no evidence of disease progression for MF patients according to IWG-MRT response criteria [2] (for criteria see Appendices 5 & 6)
- Proportion of patients in each response category according to IWG-MRT response criteria [2] for MF patients and 2013 ELN response criteria [3] for ET/PV patients at 24 weeks of treatment
- Proportion of patients showing an improvement in response category at 24 weeks compared to baseline according to 2009 ELN criteria for ET/PV patients [1] and according to IWG-MRT response criteria [2] for MF patients. Patients who are in a higher category at week 24 compared to baseline will be classed a success. Patients who enter the trial in CR and who maintain a CR will also be classed as a success in this outcome

2.2.1 Exploratory outcome measures

- Change in allele burden between weeks 12, 24 and baseline.
- Proportion of patients showing a decrease in requirement for cytoreduction at 24 weeks compared to baseline
- Proportion of patients showing a decrease in allele burden of ≥50% at 36 and 48 weeks compared to baseline
- Duration of reduction in the mutant allele burden, defined as time from first observed reduction of ≥50% until reduction from baseline becomes <25% or patients death.
- Expression (RNAseq), DNA-protein interaction (ChipSeq) and methylation studies focused on oestrogen receptor signalling in haematopoietic progenitors obtained from peripheral blood and bone marrow before (peripheral blood only) and after tamoxifen treatment

3. TRIAL DESIGN

This is a single arm, open label, multicentre, phase II trial of tamoxifen in patients with MPNs.

The aim of the study is to evaluate the activity of tamoxifen in terms of reducing allele burden and the safety of giving tamoxifen in this group of patients. A single stage A'herns design has been chosen to give evidence of activity for the treatment.

All patients will receive a minimum of 24 weeks of treatment with tamoxifen at 20mg od. Patients will be seen at weeks 4, 12, 18 and 24. Mutant allele burden of JAK2-V617F,CALR 5bp insertion (exon 9) and CALR 52bp deletion (exon 9) will be assessed at baseline. Allele burden will be reassessed at 12 weeks and 24 weeks for the mutation(s) identified at baseline. Patients may continue treatment

beyond 24 weeks at the discretion of the treating Investigator. If patients do continue treatment they should be followed up a minimum of 12 weekly and their allele burden reassessed at 36 weeks and 48 weeks as applicable. All patients will be followed up until 28 days after the last dose of tamoxifen treatment.

4. ELIGIBILITY

4.1 Inclusion Criteria

- Age ≥ 60 years (men aged between 50-59 may also be considered following discussion with the Chief Investigator)
- Women must be post-menopausal (defined as amenorrhoeic for at least 12 consecutive months following cessation of all exogenous hormonal treatments)
- Confirmed diagnosis of JAK2-V617F,CALR 5bp insertion (exon 9) or CALR 52bp deletion (exon 9) positive Essential Thrombocythaemia (ET), Polycythaemia Vera (PV) or Myelofibrosis (MF) (primary or secondary) for ≥ 6 months
- JAK2-V617F, CALR 5bp insertion (exon 9) or CALR 52bp deletion (exon 9) mutant allele burden ≥ 20% in peripheral blood granulocyte DNA at study entry (**assessed via central review**)
- WHO performance status 0-2 (see Appendix 1)
- For patients with PV or ET, maintenance of platelet count ≤600 x 10^9/L, WBC ≤25 x 10^9/L and venesection requirements ≤1 per month for the previous 3 months prior to registration, without introduction of any new therapeutic agents for their MPN for 6 months prior to registration
- For patients with MF, there must not have been any evidence of disease progression* for the previous 6 months (prior to registration) and no new therapeutic agents for their MPN introduced during this period
- Patients receiving cytoreductive therapy (with the exception of interferon alpha or investigational agents) for their MPN (not solely aspirin or venesection)
- Adequate hepatic function, defined as:
    - bilirubin ≤ 1.5 x ULN (patients with elevated bilirubin due to Gilbert's syndrome are eligible)
    - AST/ALT/ALP ≤ 2.5 x ULN
- Adequate renal function (creatinine clearance >30 mL/min)
- Male patients must agree to use effective contraception during participation in the trial and for 2 months after the last dose of trial treatment
- Patient must be able to give written informed consent

*Defined by IWG-MRT ELN criteria (Appendix 6). Please note no baseline bone marrow is required to confirm absence of "Leukemic transformation confirmed by a bone marrow blast count of ≥20%".

4.2 Exclusion Criteria

- Leukaemic transformation (>20% blasts in blood, marrow or extramedullary site).
- Accelerated phase of disease as indicated by ≥10% blasts in the peripheral blood
- Treatment of ET, PV or MF with Interferon alpha or other investigational agents for their MPN within 6 months prior to trial entry. JAK inhibitors, such as ruxolitinib, are allowed if taken continuously for ≥6 months prior to registration (dose changes during that period will be allowed)
- Any of the following previous thrombotic events at any time:
    - Portal or other splanchnic venous thrombosis
    - Vascular access complication
    - Ischemia cerebrovascular
    - Stroke
    - Transient Ischaemic attack
    - Superficial thrombophlebitis
    - Venous Thromboembolic events including pulmonary embolism (PE) and deep vein thrombosis (DVT)
    - Peripheral vascular ischemia
    - Visceral arterial ischemia
    - Acute coronary syndrome
    - Myocardial infarction

- Previous malignancy within 5 years with the exception of adequately treated cervical carcinoma in situ or localized non-melanoma skin cancer
- Previous endometrial cancer, hyperplasia or polyps
- Prior treatment with hematopoietic stem cell transplantation
- Patients who do not carry JAK-2V617F, CALR 5bp insertion (exon 9) or CALR 52bp deletion (exon 9) mutations, or whose allele burden is <20% at study entry (**assessed via central review)**
- Female patients receiving hormone replacement therapy
- Hypertriglyceridemia > grade 1
- Any serious underlying medical condition (at the judgment of the Investigator), which could impair the ability of the patient to participate in the trial (e.g. liver disease, active autoimmune disease, uncontrolled diabetes, uncontrolled infection (HIV, Hepatitis B and C), known genetic defect (apart from MPN) relating to venous thromboembolic events, or psychiatric disorder precluding understanding of trial information)
- Known hypersensitivity to tamoxifen or hypersensitivity to any other component of tamoxifen
- Concomitant drugs contraindicated for use with the trial drug according to the Summary of Product Characteristics (Appendix 8)
- Known planned scheduled elective surgery during study with the exception of dental and low risk eye surgery (e.g. cataracts)

5. SCREENING AND CONSENT

5.1 Screening

Investigators will be expected to maintain a Screening Log of all potential study candidates. This Log will include limited information about the potential candidate (e.g. date of birth and gender), the date and outcome of the screening process (e.g. enrolled into study, reason for ineligibility, or refused to participate).

For patients who appear to meet the criteria for participation in the study, the Investigator will provide information to allow them to make an informed decision regarding their participation. If informed consent is given (see section 5.2), the Investigator will conduct a full screening evaluation to ensure that the patient satisfies all inclusion and exclusion criteria. A patient who gives written informed consent and who satisfies all the inclusion and exclusion criteria may be registered onto the study. Note that assessments conducted as standard of care do not require informed consent and may be provided as screening data if conducted within the stipulated number of weeks prior to registration. Assessments required in screening are listed in the schedule of assessments and below.

5.1.1 Screening Assessments

All patients will be screened prior to registration. The following screening assessments will be performed within 6 weeks prior to trial entry unless stated otherwise (see section 7.2 for more detail):

- Collect demographic data
- Collect medical history (including prior diagnosis and treatments) with particular attention to gynaecological history
- Medical exam including height (baseline only), weight, blood pressure, WHO performance status, palpation of liver and spleen
- Ultrasound to assess spleen size
- Patient to complete MPN-SAF (Appendix 9)

Blood tests

- Full blood count and biochemistry – please see section 7.2 for full details of tests to include
- Full fasting lipid profile to include triglycerides
- Blood sample collection for **central analysis** of JAK2-V617F, CALR 5bp insertion (exon 9) and CALR 52bp deletion (exon 9) mutant allele burden. Patients must be able to start trial treatment within 6 weeks of this blood sample collection and patients cannot be registered until confirmation of eligibility has been received from the Trials Office that the patient has an allele burden ≥20%.

5.2 Informed Consent

It is the responsibility of the Investigator, as captured on the Site Signature and Delegation Log, to obtain written informed consent for each patient prior to performing any trial related procedure. A Patient Information Sheet is provided to facilitate this process. Investigators must ensure that they adequately explain the aim, trial treatment, anticipated benefits and potential hazards of taking part in the trial to the patient. The Investigator should also stress that the patient is completely free to refuse to take part or withdraw from the trial at any time. The patient should be given ample time (e.g. 24 hours) to read the Patient Information Sheet and to discuss their participation with others outside of the site research team. The patient must be given an opportunity to ask questions which should be answered to their satisfaction. The right of the patient to refuse to participate in the trial without giving a reason must be respected.

If the patient expresses an interest in participating in the trial they should be asked to sign and date the latest version of the Informed Consent Form. The Investigator or designate must then sign and date the form. A copy of the Informed Consent Form should be given to the patient, a copy should be filed in the hospital notes, and the original placed in the Investigator Site File (ISF). Once the patient is entered into the trial the patient's trial number should be entered on the Informed Consent Form maintained in the ISF. In addition, if the patient has given explicit consent a copy of the signed Informed Consent Form must be sent in the post to the Trials Office for review.

Details of the informed consent discussions should be recorded in the patient's medical notes, this should include date of, and information regarding, the initial discussion, the date consent was given, with the name of the trial and the version number of the Patient Information Sheet and Informed Consent Form. Throughout the trial the patient should have the opportunity to ask questions about the trial and any new information that may be relevant to the patient's continued participation should be shared with them in a timely manner. On occasion it may be necessary to re-consent the patient in which case the process above should be followed and the patient's right to withdraw from the trial respected. The patient should be given ample time to read changes made to the patient information sheet, which may vary depending on the nature of the change and may re-consent at the same visit that new information is provided, if they wish to do so.

Electronic copies of the Patient Information Sheet and Informed Consent Form are available from the Trials Office and should be printed or photocopied onto the headed paper of the local institution.

Details of all patients approached about the trial should be recorded on the Patient Screening/Enrolment Log and with the patient's prior consent their General Practitioner (GP) should also be informed that they are taking part in the trial. A GP Letter is provided electronically for this purpose.

6. TRIAL ENTRY

Patients will be registered to the trial via the Cancer Research UK Clinical Trials Unit (CRCTU).

Confirmation that the patient meets the inclusion criteria (≥20%) for peripheral blood mutant allele burden must have been confirmed by the Trials Office prior to registration.

Registration can be conducted via the eCRF by logging on to:

https://www.cancertrials.bham.ac.uk/TAMARINLive

Or calling ☎**: 0121 371 7866 or 0121 371 7865; 9am-5pm Monday to Friday.**

Login details will be provided by the Trials Office as part of site initiation.

An eligibility checklist and registration form (found in the ISF) should be completed prior to registration (via phone) by the Investigator or designee. If the patient is registered via the online system, the eligibility checklist should be completed prior to registration and following registration, the patient trial number will be provided and a report can be printed as a confirmation. If the patient is registered via phone, the patient trial number will be given over the telephone, followed by a fax confirmation.

7. TREATMENT DETAILS

7.1 Trial Treatment

Tamoxifen is considered an investigational medicinal product (IMP) for the purposes of the trial from the point of dispensing.

Sites are to use tamoxifen from standard hospital stock. No free or discounted product is offered as part of the trial.

Any brand of tamoxifen may be used provided it is in tablet form and administered orally.

Medication labels in English will be provided in the Pharmacy File. These must be added at the point of dispensing and the local information and patient trial number added.

Patients should be given enough tamoxifen to last until their next scheduled visit but the schedule for prescribing is at local site discretion (e.g. can be prescribed 4 weekly initially, rather than 2 weekly).

Patients will receive 20mg once daily. Patients should start trial treatment following registration but this must be within 6 weeks of the assessment of allele burden and continue daily for 24 weeks. At this point, further treatment may be prescribed if the patient is receiving benefit (at the discretion of the treating Investigator).

As guidance, treatment continuation will be encouraged but not mandated for patients who do not experience persistent side effects greater than grade 1 or thrombotic events of any grade and that fulfil one or more of the following criteria at 24 weeks:

- ≥25% reduction in allele burden compared to baseline
- Improvement of haematological response compared to baseline without changes in cytoreductive therapy dose according to 2009 ELN criteria for ET/PV patients and to IWG-MRT response criteria for MF patients
- A decrease in requirement for cytoreduction without deterioration of haematological response compared to baseline according to 2009 ELN criteria for ET/PV patients and to IWG-MRT response criteria for MF patients

7.2 Assessments

7.2.1 Demographic data and medical history

A medical history and the patient's demographics will be taken within 6 weeks of registration including the following:

- Age
- Sex
- Disease type
- Previous medical conditions (especially gynaecological)
- Previous treatments
- JAK2-V617F, CALR 5bp insertion and CALR 52bp deletion status (if known)

7.2.2 Medical examination/symptom assessment

A physical examination and symptom assessment will be assessed within 6 weeks prior to registration, at weeks 4, 12, 18, 24 and 28 days following treatment discontinuation and will include:

- WHO performance status
- Blood pressure
- Palpation of liver and spleen
- Weight

The medical examinations should also be targeted around any AEs experienced. The week 18 assessment may be performed via telephone should this be more convenient for the patient and a medical examination and haematology and biochemistry assessments are not required. This should focus on escalation or de-escalation in Tamoxifen dose, Adverse Events, Serious Adverse Events, thrombotic events, etc. However, the patient should attend this visit should there be a need for a dose escalation or de-escalation as this should be reviewed and implemented at week 18. Patients continuing with trial treatment beyond 24 weeks will receive a medical exam every 12 weeks or more frequently at local Investigator discretion.

7.2.3 Ultrasound

An ultrasound to assess spleen size will be performed within 6 weeks prior to registration and repeated at 24 weeks. For PV/ET patients entering the trial with PR and enlarged spleen, the

ultrasound at 24 weeks will be performed earlier if a CR is suspected. Thus all patients will have 2 ultrasounds in total. Paper copies of ultrasound results should be posted to the Trial Office upon receipt.

7.2.4 Haematology

A full blood count will be performed within 6 weeks prior to registration, at weeks 4, 12, 18, 24 and 28 days following treatment discontinuation and will include:

- Red blood cell (RBC count)
- Haemoglobin (Hb)
- White blood cell (WBC) count
- Lymphocytes
- Platelets
- Haematocrit (Hct)
- Absolute neutrophil count (ANC)
- Blood film

Patients continuing with trial treatment beyond 24 weeks will receive a full blood count every 12 weeks or more frequently at local investigator discretion.

7.2.5 Biochemistry

Standard biochemistry tests will be performed within 6 weeks prior to registration, at weeks 4, 12, 18, 24 and 28 days following treatment discontinuation and must include the following tests:

- Alanine Aminotransferase (ALT) or Aspartate Aminotransferase (AST)
- Lactate dehydrogenase (LDH)
- Bilirubin
- Estimated glomerular filtration rate (eGFR) or calculated creatinine clearance
- Alkaline Phosphatase (ALP)
- Calcium

Patients continuing with trial treatment beyond 24 weeks will receive biochemistry tests every 12 weeks or more frequently at local investigator discretion.

7.2.6 Lipid profile

As tamoxifen is commonly associated with elevated triglyceride levels, a full fasting lipid profile to include triglycerides will be performed at baseline, 12 weeks and 24 weeks. If triglycerides are elevated at 24 weeks and the patient is continuing therapy with tamoxifen, these levels should continue to be monitored at local clinical discretion.

7.2.7 Bone marrow evaluation

Patients will be required to undergo a bone marrow aspirate and trephine biopsy 24 weeks after starting treatment with tamoxifen. This will be assessed centrally for response using IWG-MRT Criteria (Appendix 6) for MF patients and 2013 ELN Criteria (Appendix 7) for ET/PV patients.

7.2.8 Quality of Life

All patients will be required to complete the Myeloproliferative Neoplasm Symptom Assessment Form Total Symptom Score (MPN-SAF TSS) [23] at baseline and week 24 of the study. The questionnaire can be found in Appendix 9. This will be used in order to assess symptom improvement which forms part of the response evaluation. The research team should complete the first page of the booklet prior to providing this to the patient.

7.2.9 Assessment of response

Weeks 2-16

ET and PV patients will be assessed at each protocol scheduled visit for haematological response according to 2009 ELN criteria [1] (Appendix 5) by the Investigator. This will include review of the platelet count, HCT (PV only) and WBC in combination with ultrasound (if applicable) and requirement for venesection. An ultrasound is only applicable prior to 24 weeks if the patient entered the trial in haematological PR with an enlarged spleen on imaging. In these cases, an ultrasound showing a normal spleen must be obtained in order to report haematological CR when this is suspected.

For MF patients, assessment of haematological response will be performed at each protocol scheduled visit using the IWG-MRT criteria [2] (Appendix 6). “Maintenance of response” will be defined as no evidence of disease progression. Disease progression is defined in Appendix 6 but please note a bone marrow to confirm disease progression prior to 24 weeks should only be performed if clinically indicated according to local practice.

Week 24

The trial bone marrow performed at week 24 will be centrally reviewed and a trephine histopathology report issued. Sites will be requested to provide an anonymised report from the latest bone marrow assessment available for each patient prior to registration to ensure a comparison report can be prepared from the trial biopsy at 24 weeks.

Central review of the bone marrow trephine sample will not be fed back in real time and therefore cannot be a guide for patient management.

Sites will then perform the 24 week response assessment upon receipt of this histopathology report using all the relevant clinical data.

For ET and PV patients, assessment of response at 24 weeks will be performed against the 2013 ELN criteria [3] (Appendix 7) using the BM report and results of blood counts (platelets, WBC, haematocrit), blood film, liver/spleen on palpation, symptom assessment (via MPN SAF) and occurrence of haemorrhagic or thrombotic events.

For MF patients, assessment of response at 24 weeks will be performed against IWG-MRT criteria [2] (Appendix 6) using the BM report and results of blood counts (haemoglobin, neutrophils, platelets), blood film for presence of immature myeloid cells and spleen on palpation.

7.3 Sample Collection

7.3.1 Bone marrow samples

Up to 20ml of bone marrow aspirate taken at 24 weeks should be sent to Cambridge Stem Cell Biobank (CBSB) in Lithium Heparin tubes. A trephine sample should also be sent in 1% paraformaldehyde in phosphate buffer using the container provided by the Trials Office. This trephine should represent the whole sample taken at the time of analysis and not cut or processed locally. This should be posted in a special delivery safe box to ensure it is received next day for processing at the central laboratory. Refer to the sample collection guidelines for further information.

7.3.2 Blood samples

Peripheral blood samples to allow analysis of JAK2-V617F, CALR 5bp insertion (exon 9) and CALR 52bp deletion (exon 9) mutant allele burden should be collected in lithium heparin tubes and sent to Cambridge at baseline, 12 weeks and 24 weeks.

At baseline and 24 weeks, 60 ml of blood will be collected to allow comparison of oestrogen receptor signalling in progenitor cells.

At week 12, 30 ml blood should be obtained. The remainder of the blood not required to assess allele burden will be banked at CBSB.

The level of allele burden at 12 weeks for each patient will be provided to sites in time to inform a decision about tamoxifen dose escalation and treatment continuation beyond 24 weeks. It is not possible to provide these in real time and therefore they cannot be used as a guide for patient management.

For responder patients continuing treatment beyond 24 weeks and 36 weeks, 60 ml of peripheral blood in lithium heparin tubes should be sent to the CBSB to reassess allele burden at 36 weeks and 48 weeks, respectively. The level of allele burden observed at 24, 36 and 48 weeks for each patient will also be provided to sites to inform a decision about further tamoxifen treatment continuation. It is not possible to provide these in real time and therefore they cannot be used as a guide for patient management.

In the unlikely event that a trial blood sample is lost, damaged or mishandled so it cannot be analysed, the trial office will request another sample.

7.4 Dose Modifications

Unless prevented by persistent side effects greater than grade 1, dose escalations to 40 mg tamoxifen od are encouraged, but not mandated, for patients either not showing a reduction in allele burden ≥50% or not showing an improvement of haematological response at 12 weeks compared to baseline.

Dose reductions to 10 mg tamoxifen od are allowed, but not mandated, for patients that develop mild/moderate side effects (grades 1-2) except for those experiencing thrombotic events, which will mandate permanent treatment discontinuation regardless of grade.

Dose modifications are only permitted on an individual basis at the discretion of the Chief Investigator/Clinical Coordinator. Please contact the Trials Office to discuss any dose changes. All changes must be recorded on the CRF.

7.5 Treatment Compliance

The local trial pharmacist will be responsible for maintaining and updating the drug accountability log in the pharmacy file. All unfinished packs/bottles of tamoxifen at the end of treatment/change of dose will be returned to the trial pharmacist who will count and document any unused medication. All IMP can then be destroyed in accordance with local pharmacy practice and this will be documented on the drug accountability log in the hospital pharmacy file. Refer to the Pharmacy Manual for further information.

In addition, patients will be issued with a Patient Diary at each visit as necessary for the duration of trial treatment. Patients will be asked to complete the diary, recording the time that each dose was taken, and whether any doses were missed. The diary also includes a section where the patient can record any relevant information such as side effects suffered or reasons for missed doses. The completed diary will be reviewed by the site at clinic visits and returned to the Trials Office. The patient diary will also remind patients to tell their doctor if they are receiving any other medications.

7.6 Supportive Treatment

Patients should continue to receive cytoreductive therapy for their MPN such as hydroxycarbamide or anagrelide, in addition to other supportive treatment such as venesection or aspirin as applicable. However, patients must not receive any treatments listed in section 7.7.

7.7 Concomitant Medication

Patients must not receive interferon alpha for their MPN as is known to impact in clone size. Patients can have received interferon alpha previously as long as the last dose was >6 months from trial entry.

Patients should not receive any medications contraindicated with tamoxifen according to the SPC. At the time of writing, potent inhibitors of CYP2D6 (e.g. paroxetine, fluoxetine, quinidine, cinacalcet or bupropion (refer to Appendix 8)) should be used with caution and avoided where possible.

As tamoxifen is metabolised by CYP3A4, caution is also required when co-administering with drugs such a rifampicin, and alternatives should be prescribed where possible.

In addition, tamoxifen should not be given to patients who have received it in the past and who have experienced hypersensitivity to the product or any of its ingredients.

For patients on coumarin type anticoagulants such as warfarin, an increase in anticoagulant effect can occur with tamoxifen and therefore additional monitoring is recommended.

7.8 Patient Follow Up

All patients will be followed up until 28 days after the last administration of tamoxifen. This applies if treatment is stopped at 24 weeks, or 28 days after the last treatment if the patient continues beyond 24 weeks. Patients stopping trial treatment early will continue to be followed up for the trial outcomes as per protocol schedule unless the patient specifically withdraws consent.

If patients discontinue study drug prior to 24 weeks, collection of blood samples for central assessment of mutant allele burden should continue to be taken at the protocol specified time points providing the patient received at least 2 months of tamoxifen treatment, and the time point is <3 months from the last date of trial treatment.

Patients continuing trial treatment beyond 24 weeks will have their mutant allele burden reassessed at 36 weeks. Patients continuing trial treatment beyond 36 weeks will have their mutant allele burden reassessed at 48 weeks.

7.9 Patient Withdrawal

In the event of discontinuation of study treatment, e.g. unacceptable toxicity or patient choice, full details of the reason/s for discontinuation should be recorded on the CRF.

All patients, including non-compliant subjects, should be followed up according to the protocol schedule unless they withdraw specific consent.

In the event of a patient's decision to withdraw from the trial, the Investigator should ascertain from which aspects of the trial the patient wishes to withdraw and record the details on the appropriate CRF. All information and blood/bone marrow samples collected up until the point of retraction will be retained and analysed. If a patient chooses to withdraw from treatment only, the patient should discontinue treatment and continue to be assessed in accordance with the protocol. If a patient wishes to withdraw from the trial (i.e. including trial specific assessments), but is willing for further data to be supplied to the Trials Office, then further routine 'follow-up' data (e.g. toxicity data) will continue to be supplied by the Investigator to the Trials Office.

Patients who stop treatment due to adverse experiences (clinical or laboratory) will be treated and followed according to accepted medical practice. All pertinent information concerning the outcome of such treatment must be recorded in the CRF.

The following are justifiable reasons for the Investigator to stop study treatment:

- Unforeseen events: any event which in the judgement of the Investigator makes further treatment inadvisable
- Serious violation of the trial protocol (including persistent patient non-attendance and persistent non-compliance)
- Stopping by the Investigator for clinical reasons not related to the study drug treatment

Patients must stop study treatment in the event of:

- Unacceptable toxicity, defined as:
    - Grade 3-4 adverse event considered related to the trial IMP
    - Persisting grade 1-2 adverse events considered related to the trial IMP lasting more than 14 days and that do not resolve within 4 weeks of reducing tamoxifen to 10 mg od
- Thrombotic event of any grade
- SAE requiring permanent discontinuation of treatment
- Transformation to MDS or AML

8. ADVERSE EVENT REPORTING

The collection and reporting of Adverse Events (AEs) will be in accordance with the Medicines for Human Use Clinical Trials Regulations 2004 and its subsequent amendments. Definitions of different types of AE are listed in Appendix 3. The Investigator should assess the seriousness and causality (relatedness) of all AEs experienced by the patient (this should be documented in the source data) with reference to the SPC.

8.1 Reporting Requirements

8.1.1 Adverse Events

All medical occurrences which meet the definition of an AE (see Appendix 3 for definition) should be reported. Please note this does not include abnormal laboratory findings. An abnormal laboratory value is only considered to be an AE if the abnormality:

- Results in early discontinuation from the study treatment and/or
- Requires study drug dose modification or interruption, any other therapeutic intervention or is judged to be of significant clinical importance

If a laboratory abnormality is one component of a diagnosis or syndrome, then only the diagnosis or syndrome should be recorded.

Pre-existing symptoms present prior to commencing trial treatment should only be reported as an AE if they increase in grade.

Details of all AEs experienced by the patient should be recorded in the hospital notes.

AEs should be graded according to Common Terminology Criteria for Adverse Events (CTCAE) v4.0 criteria and relatedness to the study IMP should be assessed by the Investigator. All AEs should be recorded via the running Adverse Events Form throughout the study. Where the patient experiences a change in grade of an AE, each change in grade should be recorded as a new AE.

8.1.2 Serious Adverse Events

Investigators should report all AEs that meet the definition of an SAE (see Appendix 3 for definition).

8.1.2.1 Monitoring pregnancies for potential Serious Adverse Events

It is important to monitor the outcome of pregnancies of patient's partners in order to provide SAE data on congenital anomalies or birth defects.

In the event that a patient's partner becomes pregnant during the SAE reporting period please complete a Pregnancy Notification Form (providing the patient's details) and return to the Trials Office as soon as possible. The patient should be given a Release of Medical Information Form to give to their partner. If the partner is happy to provide information on the outcome of their pregnancy they should sign the Release of Medical Information Form. Once consent has been obtained provide details of the outcome of the pregnancy on a follow-up Pregnancy Notification Form and if necessary also complete an SAE Form.

8.1.3 Reporting period

Details of all AEs (except those listed above) will be documented and reported from the date of commencement of protocol defined treatment until 28 days after the administration of the last treatment. SAEs that are judged to be at least possibly related to tamoxifen must still be reported in an expedited manner irrespective of how long after IMP administration the reaction occurred. Special attention should be paid to women who develop endometrial problems.

8.2 Reporting Procedure

8.2.1 Site

8.2.1.1 Adverse Events

AEs should be reported on an AE Form (and where applicable on an SAE Form). The AE Form is a continuous form completed via the eCRF. The form should be checked and updated as applicable at each visit.

AEs will be reviewed using the CTCAE version 4.0 (see Appendix 4). Any AEs experienced by the patient but not included in the CTCAE should be graded by an Investigator and recorded on the AE Form using a scale of (1) mild, (2) moderate or (3) severe. For each sign/symptom, the highest grade observed since the last visit should be recorded.

8.2.1.2 Serious Adverse Events

For more detailed instructions on SAE reporting refer to the SAE Form Completion Guidelines contained in the Investigator Site File (ISF).

AEs defined as serious and which require reporting as an SAE (excluding events listed in Section 8.1 above) should be reported on an SAE Form. When completing the form, the Investigator will be asked to define the causality and the severity of the AE which should be documented using the CTCAE version 4.0.

On becoming aware that a patient has experienced an SAE, the Investigator (or delegate) must complete, date and sign an SAE Form. The form should be faxed together with a SAE Fax Cover Sheet to the Trials Office using one of the numbers listed below as soon as possible and no later than 24 hours after first becoming aware of the event:

To report an SAE, either e-mail or fax the SAE Form with an SAE Fax Cover Sheet to:

0121 371 7874 or 0121 371 4398 <u>Or</u> Tamarin@trials.bham.ac.uk

On receipt the Trials Office will allocate each SAE a unique reference number. This number will be transcribed onto the SAE Fax Cover Sheet which will then be faxed or e-mailed back to the site as proof of receipt. If confirmation of receipt is not received within 1 working day please contact the Trials Office. The SAE reference number should be quoted on all correspondence and follow-up reports regarding the SAE. The SAE Fax Cover Sheet completed by the Trials Office should be filed with the SAE Form in the ISF.

For SAE Forms completed by someone other than the Investigator the Investigator will be required to countersign the original SAE Form to confirm agreement with the causality and severity assessments. The form should then be returned to the Trials Office in the post and a copy kept in the ISF.

Investigators should also report SAEs to their own Trust in accordance with local practice.

8.2.1.3 Provision of follow-up information

Patients should be followed up until resolution or stabilisation of the event. Follow-up information should be provided on a new SAE Form (refer to the SAE Form Completion Guidelines for further information).

8.2.2 Trials Office

On receipt of an SAE Form seriousness and causality will be determined independently by a Clinical Coordinator. An SAE judged by the Investigator or Clinical Coordinator to have a reasonable causal relationship with the trial medication will be regarded as a Serious Adverse Reaction (SAR). The Clinical Coordinator will also assess all SARs for expectedness. If the event meets the definition of a SAR that is unexpected (i.e. is not defined in the Reference Safety Information (RSI)) it will be classified as a Suspected Unexpected Serious Adverse Reaction (SUSAR).

8.2.3 Reporting to the Competent Authority and main Research Ethics Committee

8.2.3.1 Suspected Unexpected Serious Adverse Reactions

The Trials Office will report a minimal data set of all individual events categorised as a fatal or life threatening SUSAR to the Medicines and Healthcare products Regulatory Agency (MHRA) and main Research Ethics Committee (REC) within 7 days. Detailed follow-up information will be provided within an additional 8 days.

All other events categorised as SUSARs will be reported within 15 days.

8.2.3.2 Serious Adverse Reactions

The Trials Office will report details of all SARs (including SUSARs) to the MHRA and main REC annually from the date of the Clinical Trial Authorisation, in the form of a Development Safety Update Report (DSUR).

8.2.3.3 Adverse Events

Details of all AEs will be reported to the MHRA on request.

8.2.3.4 Other safety issues identified during the course of the trial

The MHRA and main REC will be notified immediately if a significant safety issue is identified during the course of the trial.

8.2.4 Investigators

Details of all SUSARs and any other safety issue which arises during the course of the trial will be reported to Principal Investigators. A copy of any such correspondence should be filed in the ISF.

8.2.5 Trial Steering Committee

The independent Trial Steering Committee (TSC) will review all SAEs.

9. DATA HANDLING AND RECORD KEEPING

9.1 Data Collection

The electronic Case Report Form (eCRF) will comprise, but is not limited to, the following forms:

Form	Summary of data recorded	Schedule for submission
Eligibility Checklist	Confirmation of eligibility	Completed prior to registration

Registration	Patient details	Completed as part of registration
Baseline	Medical history, previous treatment, baseline characteristics, haematology/biochemistry assessments and details of medical exam	Within 2 weeks of registration
Treatment Form	Treatment details, haematology/biochemistry assessments and details of medical exam	Within 2 weeks of scheduled visit
Treatment Form – Week 24	Treatment details, haematology/biochemistry assessments, details of medical exam and bone marrow assessment	Within 2 weeks of scheduled visit
28 Days post Treatment Follow-up Form	Haematology/biochemistry assessments, details of medical exam	Within 2 weeks of scheduled visit
Adverse Event Form	Details of AEs experienced	Continuous
Concomitant Medication Form	Details of concomitant medications received	Continuous
Treatment continuation Form	Treatment details, haematology/biochemistry assessments and details of medical exam for patients continuing treatment beyond 24 weeks	Within 2 weeks of visit
Treatment Discontinuation Form	Date and reason for premature stopping of treatment	Within 2 weeks of stopping treatment
Death Form	Date and cause of death	Immediately upon notification of patient's death
Deviation Form	Completed in the event of a deviation from the protocol	Immediately upon discovering deviation
Withdrawal Form	Used to notify the Trials Office of patient withdrawal from the trial	Immediately upon patient withdrawal
Event Form	Completed when a patient experiences a thromboembolic event of any grade	Within 2 weeks of resolution

Ad hoc forms

Serious Adverse Event Form

Pregnancy Notification Form

This trial will use an electronic remote data capture (eRDC) system which will be used for completion of CRFs.

Access to the eRDC system will be granted to individuals via the Trials Office. SAE reporting and Notification of Pregnancy will continue to be paper-based.

The CRF must be completed by the Investigator or an authorised member of the site research team (as delegated on the Site Signature and Delegation Log) within specified timeframes (listed above and found in the eRDC completion guidelines). The exceptions to this are the SAE Form which must be reviewed and co-signed by the Investigator.

Data reported on each form should be consistent with the source data or the discrepancies should be explained. If information is not known, this must be clearly indicated on the form by selecting e.g. "Not done" where available. Please note this option may not be available for Critical Data Items which are essential to the analysis. All missing and ambiguous data will be queried. All sections are to be completed before returning.

In all cases it remains the responsibility of the Investigator to ensure that the CRF has been completed correctly and that the data are accurate.

Trial forms on the eRDC system may be amended by the Trials Office, as appropriate, throughout the duration of the trial. This will not constitute a protocol amendment.

9.2 Archiving

It is the responsibility of the Principal Investigator to ensure all essential trial documentation and source records (e.g. signed Informed Consent Forms, Investigator Site Files, Pharmacy Files, patients' hospital notes etc.) at their site are securely retained for at least 15 years after the end of the trial or following the processing of all biological material collected for research, whichever is the later. Do not destroy any documents without prior approval from the CRCTU Document Storage Manager.

10. QUALITY MANAGEMENT

10.1 Site Set-up and Initiation

All sites will be required to sign a Clinical Study Site Agreement prior to participation. In addition all participating Investigators will be asked to sign the necessary documents e.g. registration forms, PI Protocol Signature Pages and supply a current CV and evidence of GCP training within the last 2 years to the Trials Office. All members of the site research team will also be required to sign the Site Signature and Delegation Log, which should be returned to the Trials Office. Prior to commencing recruitment all sites will undergo a process of initiation. Key members of the site research team will be required to attend either a meeting or a teleconference covering aspects of the trial design, protocol procedures, AE reporting, collection and reporting of data and record keeping. Sites will be provided with an ISF and a Pharmacy File containing essential documentation, instructions, and other documentation required for the conduct of the trial. The Trials Office must be informed immediately of any change in the site research team.

10.2 On-site Monitoring

Monitoring will be carried out as required following a risk assessment and as documented in the CRCTU Quality Management Plan. Additional on-site monitoring visits may be triggered for example by poor CRF return, poor data quality, low SAE reporting rates, excessive number of patient withdrawals or deviations. If a monitoring visit is required the Trials Office will contact the site to arrange a date for the proposed visit and will provide the site with written confirmation. Investigators will allow the TAMARIN trial staff access to source documents as requested.

10.3 Central Monitoring

Where a patient has given explicit consent sites are requested to send in copies of signed Informed Consent Forms for in-house review.

Trials staff will be in regular contact with the site research team to check on progress and address any queries that they may have. Trials staff will check completed CRFs for compliance with the protocol, data consistency, missing data and timing. Sites will be sent Data Clarification Forms via the eRDC system requesting missing data or clarification of inconsistencies or discrepancies.

Sites may be suspended from further recruitment in the event of serious and persistent non-compliance with the protocol and/or GCP, and/or poor recruitment. Any major problems identified during monitoring may be reported to Trial Management Group (TMG) and Trial Steering Committee (TSC) and the relevant regulatory bodies. This includes reporting serious breaches of GCP and/or the trial protocol to the main REC) and the MHRA.

10.4 Audit and Inspection

The Investigator will permit trial-related monitoring, audits, ethical review, and regulatory inspection(s) at their site, providing direct access to source data/documents.

Sites are also requested to notify the Trials Office of any MHRA inspections.

10.5 Notification of Serious Breaches

In accordance with Regulation 29A of the Medicines for Human Use (Clinical Trials) Regulations 2004 and its amendments the Sponsor of the trial is responsible for notifying the licensing authority in writing of any serious breach of:

- The conditions and principles of GCP in connection with that trial or;

- The protocol relating to that trial, within 7 days of becoming aware of that breach

For the purposes of this regulation, a "serious breach" is a breach which is likely to effect to a significant degree:

- The safety or physical or mental integrity of the subjects of the trial; or
- The scientific value of the trial

Sites are therefore requested to notify the Trials Office of a suspected trial-related serious breach of GCP and/or the trial protocol. Where the Trials Office is investigating whether or not a serious breach has occurred sites are also requested to cooperate with the Trials Office in providing sufficient information to report the breach to the MHRA where required and in undertaking any corrective and/or preventive action.

11. END OF TRIAL DEFINITION

The end of trial will be the last patient's last visit. The Trials Office will notify the MHRA and main REC that the trial has ended and will provide them with a summary of the clinical trial report within 12 months of the end of trial.

12. STATISTICAL CONSIDERATIONS

12.1 Definition of Outcome Measures

12.1.1 Primary outcome measure

The primary outcome for the trial is the number of patients who experience a reduction of ≥50% in peripheral blood JAK2-V617F,CALR 5bp insertion (exon 9) or CALR 52bp deletion (exon 9) mutant allele burden at 24 weeks. The primary outcome will be assessed centrally at University of Cambridge. Peripheral blood samples will be sent for patients at baseline and 24 weeks (+/- 4 weeks) which will be compared in parallel for JAK2-V617F or CALR 5bp insertion (exon 9) or CALR 52bp deletion (exon 9) mutant allele burden. If there is a reduction of ≥50% at 24 weeks compared to baseline for either JAK2-V617F or CALR 5bp insertion (exon 9) or CALR 52bp deletion (exon 9), the patient will have met the primary outcome.

12.1.2 Secondary outcome measures

- Proportion of patients with a reduction in the peripheral blood JAK2-V617F, CALR 5bp insertion (exon 9), or CALR 52bp deletion (exon 9) mutant allele burden of ≥50% at 12 weeks
- Toxicity measured as the number of grade 3 and 4 adverse events reported.
- The number of thrombotic events of any grade reported and validated.
- Haematological response, assessed at weeks 12 and 24, for patients who enter the study in response (CR or PR). Haematological response is defined according to 2009 ELN criteria for ET/PV patients and no evidence of disease progression for MF patients according to IWG-MRT response criteria (for criteria see Appendices 5 & 6).
- Proportion of patients in each response category according to IWG-MRT response criteria for MF patients and 2013 ELN response criteria for ET/PV patients at 24 weeks of treatment.
- Proportion of patients showing an improvement in response category at 24 weeks compared to baseline according to 2009 ELN criteria for ET/PV patients [1] and according to IWG-MRT response criteria [2] for MF patients. Patients who are in a higher category at week 24 compared to baseline will be classed a success. Patients who enter the trial in CR and who maintain a CR will also be classed as a success in this outcome.

12.1.3 Exploratory outcome measure

- Change in JAK2-V617F, CALR 5bp insertion (exon 9), or CALR 52bp deletion (exon 9) allele burden between weeks 12, 24 and baseline.
- Proportion of patients showing a decrease in requirement for cytoreduction at 24 weeks compared to baseline
- Proportion of patients showing a decrease in the peripheral blood JAK2-V617F, CALR 5bp insertion (exon 9), or CALR 52bp deletion (exon 9) mutant allele burden of ≥50% at 36 and 48 weeks compared to baseline

- Duration of reduction in the peripheral blood JAK2-V617F, CALR 5bp insertion (exon 9), or CALR 52bp deletion (exon 9) mutant allele burden, defined as time from first observed reduction of ≥50% until reduction from baseline becomes <25% or patient death.
- The expression (RNAseq), DNA-protein interaction (CHIP-Seq) and methylation studies focused on oestrogen receptor signalling in haematopoietic progenitors will be performed in the lab of Dr Mendez-Ferrer at the University of Cambridge following the collection of peripheral blood (baseline and 24 weeks, plus 36 weeks and 48 weeks if patients continue treatment beyond 24 weeks as applicable) and bone marrow aspirate samples (24 weeks only).

12.2 Analysis of Outcome Measures

12.2.1 Primary outcome measure

Analysis of the primary outcome will be completed on an intention to treat basis. The proportion of patients who have achieved the primary outcome of a reduction in allele burden of ≥50% will be presented. Given 3 or more patients have achieved this level of reduction in allele burden the treatment will be deemed to show evidence of activity and will warrant further investigation. Patients who die or are lost to follow up prior to the primary end point of 24 weeks will be classed as non-responders.

12.2.2 Secondary outcome measures

All secondary outcomes will be analysed in a descriptive manner on an intention to treat basis with no formal hypothesis testing taking place.

- The reduction in the allele burden of ≥50% at 12 weeks will also be obtained following analysis of peripheral blood at University of Cambridge. Counts and percentages of patients who have achieved each level of reduction at 12 weeks will be presented. Patients who die or are lost to follow up prior to 12 weeks will be classed as non-responders.
- The total number of grade 3 and 4 adverse events reported will be presented as well as the number of patients who have experienced one or more of these events.
- The total number of thrombotic events (of all grades) will be reported as well as the number of patients who have experienced one or more of these events.
- Haematological response at weeks 12 and 24 will be tabulated and compared using a chi^2 test or Fisher's exact test if the first is not considered appropriate.
- Proportion of patients in each response category at 24 weeks of treatment will be presented as the number and percentage of patients in each category.
- Proportion of patients showing an improvement in response category at 24 weeks will be presented as the number and percentage of patients who have shown an improvement (or maintained a CR).

12.2.3 Exploratory outcome measure

All exploratory outcomes will be analysed in a descriptive manner on an intention to treat basis with no formal hypothesis testing taking place.

- Change in allele burden at 12 and 24 weeks compared to baseline will be obtained following analysis of peripheral blood at University of Cambridge and will be presented as the median change from baseline at each time point with the interquartile ranges.
- Proportion of patients showing a decrease in requirement for cytoreduction therapy at 24 weeks compared to baseline will be presented as the number and percentage of patients who have a decrease in treatment dose. Patients who die or are lost to follow up prior to week 24 will be classed as non-responders.
- Proportion of patients showing a decrease in allele burden of ≥50% at 36 and 48 weeks compared to baseline will also be obtained following analysis of peripheral blood at University of Cambridge. Counts and percentages of patients who have achieved the reduction at 36 and 48 weeks will be presented. This will only be presented for patients who remain on treatment post week 24 and are assessed at week 36 and week 48, respectively. .
-
- Duration of reduction in the peripheral blood JAK2-V617F, CALR 5bp insertion (exon 9), or CALR 52bp deletion (exon 9) mutant allele burden, defined as time between visits from first

observed reduction of ≥50% until the visit at which reduction from baseline becomes <25% or the last visit before the patient dies, will be tabulated.
- Expression (RNAseq), DNA-protein interaction (ChipSeq) and methylation studies will be analysed and presented by the University of Cambridge.

12.3 Planned Sub Group Analyses

Due to the lack of statistical power for subgroup analyses in this early phase II trial, results provided will be exploratory only. Therefore, results should not be over interpreted and instead used as a guide for further subgroup analyses in a larger phase III setting. Subgroups to be studied include:

- Results by mutation type (JAK2-V617F, CALR 5bp insertion (exon 9), CALR 52bp deletion (exon 9))
- Primary disease (ET, PV, MF)
- Oestrogen receptor data
- Sex

12.4 Planned Interim Analysis

Accumulating data will be analysed and presented on a regular basis (6 monthly) to an independent TSC for monitoring of safety and futility. Analysis will also be performed and presented to the TSC if 7 or more patients out of the first 21 patients registered are deemed to not have tolerated treatment. A patient is classed as not being able to tolerate treatment if they discontinue treatment due to:

- Unacceptable toxicity, defined as:
  - Grade 3-4 adverse event considered related to tamoxifen
  - Persisting grade 1-2 adverse events considered related to tamoxifen lasting more than 14 days and that do not resolve within 4 weeks of reducing tamoxifen to 10 mg od
- Thrombotic event of any grade

12.5 Planned Final Analyses

The main analysis of the trial will take place once the final patient reaches all trial endpoint at week 48 or discontinues trial treatment, whatever happens first.

12.6 Power Calculations

A single stage A'herns design is used to give evidence of activity for the treatment. It is anticipated that no patients on the current treatment will achieve a reduction of ≥50% in allele burden. Given this, if 10% of the patients in this trial were to achieve the required reduction then the treatment would warrant further investigation in a randomised trial. If just 2% of the patients in the trial were to achieve the required level of reduction then the treatment would not be deemed to show any activity that would warrant further investigation. Based on these levels of acceptability and given a one sided alpha of 0.1 and power of 80%, 42 patients would be required for the study. If 3 or more of the recruited patients were to achieve a reduction in allele burden of ≥50% then it can be concluded that the treatment shows a signal of activity and the treatment warrants further investigation in a randomised trial. Patients who die or are lost to follow up prior to the response time point of 24 weeks will be classed as non-responders.

13. TRIAL ORGANISATIONAL STRUCTURE

13.1 Sponsor

The trial is sponsored by the University of Birmingham.

13.2 Coordinating Centre

The trial is being conducted under the auspices of the Cancer Research UK Clinical Trials Unit (CRCTU), University of Birmingham according to their local procedures.

13.3 Trial Management Group

The TMG will consist of the Chief Investigator, Clinical coordinator (s)/Co-Investigators, Trial Statistician and the trials team at the CRCTU. The TMG will provide overall supervision of the trial; in particular clinical set-up, ongoing management, adherence to the protocol, consideration of new

information and interpretation of the results. The TMG will meet every two months. An emergency meeting may be convened if a significant issue is identified

13.4 Trial Steering Committee

Data analyses will be supplied in confidence to an independent TSC which will be asked to give advice on whether the accumulated data from the trial, together with the results from other relevant research, justifies the continuing recruitment of further patients. During the recruitment phase of the trial the TSC is scheduled to meet 6 monthly. Additional meetings may be called if recruitment is much faster than anticipated and the TSC may, at their discretion, request to meet more frequently or continue to meet following completion of recruitment. An emergency meeting may also be convened if a safety issue is identified. The TSC would also be convened to monitor the safety of the trial in mind of the efficacy if 7 out of the first 21 patients registered to the trial do not tolerate treatment. See section 12.4.

The TSC will report directly to the Trial Management Group (TMG) who will convey the findings to the Sponsor, MHRA, REC or funder if applicable. The TSC may consider recommending the discontinuation of the trial if the recruitment rate or data quality are unacceptable or if any issues are identified which may compromise patient safety.

13.5 Finance

This is a clinician-initiated and clinician-led trial funded by Bloodwise as part of the Trials Acceleration Programme (TAP). No arrangements have been made for free or discounted tamoxifen. As the bone marrow assessment at 24 weeks could be considered a research cost, funding has been allocated to cover this and will be detailed in the Clinical Study Site Agreement. Funding has also been allocated to contribute towards site archiving costs.

No individual per patient payment will be made to NHS Trusts (other than the bone marrow research cost), Investigators or patients.

This trial has been adopted into the NIHR CRN Portfolio.

14. ETHICAL CONSIDERATIONS

The trial will be performed in accordance with the recommendations guiding physicians in biomedical research involving human subjects, adopted by the 18th World Medical Association General Assembly, Helsinki, Finland, June 1964, amended at the 48th World Medical Association General Assembly, Somerset West, Republic of South Africa, October 1996 (http://www.wma.net/en/30publications/10policies/b3/index.html).

The trial will be conducted in accordance with the Research Governance Framework for Health and Social Care, the applicable UK Statutory Instruments, (which include the Medicines for Human Use Clinical Trials 2004 and subsequent amendments and the General Data Protection Regulations and Data Protection Act 2018 and Human Tissue Act 2008) and Good Clinical Practice (GCP). This trial will be carried out under a Clinical Trial Authorisation in accordance with the Medicines for Human Use Clinical Trials regulations. The protocol will be submitted to and approved by the main Research Ethics Committee prior to circulation.

Before any patients are enrolled into the trial, the Principal Investigator at each site is required to obtain local R&D approval. Sites will not be permitted to enrol patients until written confirmation of R&D approval is received by the Trials Office.

It is the responsibility of the Principal Investigator to ensure that all subsequent amendments gain the necessary local approval. This does not affect the individual clinicians' responsibility to take immediate action if thought necessary to protect the health and interest of individual patients.

15. CONFIDENTIALITY AND DATA PROTECTION

Personal data recorded on all documents will be regarded as strictly confidential and will be handled and stored in accordance with the General Data Protection Regulation and the Data Protection Act (2018). With the patient's consent, their initials and date of birth will be collected at trial entry. Patients will be identified using only their unique registration number and date of birth on the correspondence between the Trials Office and the participating sites. However patients are asked to give permission for the Trials Office to be sent a copy of their signed Informed Consent Form which will not be anonymised. This will be used to perform in-house monitoring of the consent process.

The Investigator must maintain documents not for submission to the Trials Office (e.g. Patient Identification Logs) in strict confidence. In the case of specific issues and/or queries from the regulatory authorities, it will be necessary to have access to the complete trial records, provided that patient confidentiality is protected.

The Trials Office will maintain the confidentiality of all patient's data and will not disclose information by which patients may be identified to any third party other than those directly involved in the treatment of the patient. Representatives of the TAMARIN trial team may be required to have access to patient's notes for quality assurance purposes but patients should be reassured that their confidentiality will be respected at all times.

16. INSURANCE AND INDEMNITY

University of Birmingham employees are indemnified by the University insurers for negligent harm caused by the design or co-ordination of the clinical trials they undertake whilst in the University's employment.

In terms of liability at a site, NHS Trust and non-Trust hospitals have a duty to care for patients treated, whether or not the patient is taking part in a clinical trial. Compensation is therefore available via NHS indemnity in the event of clinical negligence having been proven.

The University of Birmingham cannot offer indemnity for non-negligent harm. The University of Birmingham is independent of any pharmaceutical company, and as such it is not covered by the Association of the British Pharmaceutical Industry (ABPI) guidelines for patient compensation.

17. PUBLICATION POLICY

Results of this trial will be submitted for publication in a peer reviewed journal. The manuscript will be prepared by the TMG and authorship will be determined in accordance with the TAP authorship policy.

Any secondary publications and presentations prepared by Investigators must be reviewed by the TMG. Manuscripts must be submitted to the TMG in a timely fashion and in advance of being submitted for publication, to allow time for review and resolution of any outstanding issues. Authors must acknowledge that the trial was performed with the support of University of Birmingham and in line with the TAP authorship policy. Intellectual property rights will be addressed in the Clinical Study Site Agreement between Sponsor and site.

18. REFERENCE LIST

1. Barosi, G., et al., *Response criteria for essential thrombocythemia and polycythemia vera: result of a European LeukemiaNet consensus conference.* Blood, 2009. **113**(20): p. 4829-33.
2. Tefferi, A., et al., *Revised response criteria for myelofibrosis: International Working Group-Myeloproliferative Neoplasms Research and Treatment (IWG-MRT) and European LeukemiaNet (ELN) consensus report.* Blood, 2013. **122**(8): p. 1395-8.
3. Barosi, G., et al., *Revised response criteria for polycythemia vera and essential thrombocythemia: an ELN and IWG-MRT consensus project.* Blood, 2013. **121**(23): p. 4778-81.
4. Smith, A., et al., *Incidence of haematological malignancy by sub-type: a report from the Haematological Malignancy Research Network.* Br J Cancer, 2011. **105**(11): p. 1684-92.
5. Finazzi, G. and T. Barbui, *Evidence and expertise in the management of polycythemia vera and essential thrombocythemia.* Leukemia, 2008. **22**(8): p. 1494-502.
6. Finazzi, G. and T. Barbui, *How I treat patients with polycythemia vera.* Blood, 2007. **109**(12): p. 5104-11.
7. James, C., et al., *A unique clonal JAK2 mutation leading to constitutive signalling causes polycythaemia vera.* Nature, 2005. **434**(7037): p. 1144-8.
8. Campbell, P.J., et al., *V617F mutation in JAK2 is associated with poorer survival in idiopathic myelofibrosis.* Blood, 2006. **107**(5): p. 2098-100.
9. Dahabreh, I.J., et al., *Is JAK2 V617F mutation more than a diagnostic index? A meta-analysis of clinical outcomes in essential thrombocythemia.* Leuk Res, 2009. **33**(1): p. 67-73.

10. Passamonti, F., et al., *A prospective study of 338 patients with polycythemia vera: the impact of JAK2 (V617F) allele burden and leukocytosis on fibrotic or leukemic disease transformation and vascular complications.* Leukemia, 2010. **24**(9): p. 1574-9.
11. Alvarez-Larran, A., et al., *JAK2V617F monitoring in polycythemia vera and essential thrombocythemia: clinical usefulness for predicting myelofibrotic transformation and thrombotic events.* Am J Hematol, 2014. **89**(5): p. 517-23.
12. Coucelo, M., et al., *JAK2V617F allele burden is associated with thrombotic mechanisms activation in polycythemia vera and essential thrombocythemia patients.* Int J Hematol, 2014. **99**(1): p. 32-40.
13. Shirane, S., et al., *Consequences of the JAK2V617F allele burden for the prediction of transformation into myelofibrosis from polycythemia vera and essential thrombocythemia.* Int J Hematol, 2015. **101**(2): p. 148-53.
14. Klampfl, T., et al., *Somatic mutations of calreticulin in myeloproliferative neoplasms.* N Engl J Med, 2013. **369**(25): p. 2379-90.
15. Nangalia, J., et al., *Somatic CALR mutations in myeloproliferative neoplasms with nonmutated JAK2.* N Engl J Med, 2013. **369**(25): p. 2391-405.
16. Sanchez-Aguilera, A., et al., *Estrogen signaling selectively induces apoptosis of hematopoietic progenitors and myeloid neoplasms without harming steady-state hematopoiesis.* Cell Stem Cell, 2014. **15**(6): p. 791-804.
17. Agrawal, S., et al., *DNA methylation of tumor suppressor genes in clinical remission predicts the relapse risk in acute myeloid leukemia.* Cancer Res, 2007. **67**(3): p. 1370-7.
18. Herman, J.G., et al., *Hypermethylation-associated inactivation indicates a tumor suppressor role for p15INK4B.* Cancer Res, 1996. **56**(4): p. 722-7.
19. Hess, C.J., et al., *Concurrent methylation of promoters from tumor associated genes predicts outcome in acute myeloid leukemia.* Leuk Lymphoma, 2008. **49**(6): p. 1132-41.
20. Yao, J., et al., *Methylation status of oestrogen receptor alpha-A: a predictor of prognosis in leukaemias.* Biosci Rep, 2010. **30**(4): p. 217-22.
21. Blum, W., et al., *Phase I study of decitabine alone or in combination with valproic acid in acute myeloid leukemia.* J Clin Oncol, 2007. **25**(25): p. 3884-91.
22. Barosi, G., et al., *Clinical end points for drug treatment trials in BCR-ABL1-negative classic myeloproliferative neoplasms: consensus statements from European LeukemiaNET (ELN) and Internation Working Group-Myeloproliferative Neoplasms Research and Treatment (IWG-MRT).* Leukemia, 2015. **29**(1): p. 20-6.
23. Emanuel, R.M., et al., *Myeloproliferative neoplasm (MPN) symptom assessment form total symptom score: prospective international assessment of an abbreviated symptom burden scoring system among patients with MPNs.* J Clin Oncol, 2012. **30**(33): p. 4098-103.

APPENDIX 1 – WHO PERFORMANCE STATUS

Grade	Explanation of activity
0	Fully active, able to carry on all pre-disease performance without restriction
1	Restricted in physically strenuous activity but ambulatory and able to carry out work of a light or sedentary nature, e.g., light house work, office work
2	Ambulatory and capable of all selfcare but unable to carry out any work activities. Up and about more than 50% of waking hours
3	Capable of only limited selfcare, confined to bed or chair more than 50% of waking hours
4	Completely disabled. Cannot carry on any selfcare. Totally confined to bed or chair
5	Dead

APPENDIX 2 - WMA DECLARATION OF HELSINKI

WORLD MEDICAL ASSOCIATION DECLARATION OF HELSINKI
Recommendations guiding physicians
in biomedical research involving human subjects
Adopted by the 18th World Medical Assembly
Helsinki, Finland, June 1964
and amended by the
29th World Medical Assembly, Tokyo, Japan, October 1975
35th World Medical Assembly, Venice, Italy, October 1983
41st World Medical Assembly, Hong Kong, September 1989
and the
48th General Assembly, Somerset West, Republic of South Africa, October 1996

INTRODUCTION

It is the mission of the physician to safeguard the health of the people. His or her knowledge and conscience are dedicated to the fulfillment of this mission.

The Declaration of Geneva of the World Medical Association binds the physician with the words, "The Health of my patient will be my first consideration," and the International Code of Medical Ethics declares that, "A physician shall act only in the patient's interest when providing medical care which might have the effect of weakening the physical and mental condition of the patient."

The purpose of biomedical research involving human subjects must be to improve diagnostic, therapeutic and prophylactic procedures and the understanding of the aetiology and pathogenesis of disease.

In current medical practice most diagnostic, therapeutic or prophylactic procedures involve hazards. This applies especially to biomedical research.

Medical progress is based on research which ultimately must rest in part on experimentation involving human subjects.

In the field of biomedical research a fundamental distinction must be recognized between medical research in which the aim is essentially diagnostic or therapeutic for a patient, and medical research, the essential object of which is purely scientific and without implying direct diagnostic or therapeutic value to the person subjected to the research.

Special caution must be exercised in the conduct of research which may affect the environment, and the welfare of animals used for research must be respected.

Because it is essential that the results of laboratory experiments be applied to human beings to further scientific knowledge and to help suffering humanity, the World Medical Association has prepared the following recommendations as a guide to every physician in biomedical research involving human subjects. They should be kept under review in the future. It must be stressed that the standards as drafted are only a guide to physicians all over the world. Physicians are not relieved from criminal, civil and ethical responsibilities under the laws of their own countries.

I. BASIC PRINCIPLES

1. Biomedical research involving human subjects must conform to generally accepted scientific principles and should be based on adequately performed laboratory and animal experimentation and on a thorough knowledge of the scientific literature.

2. The design and performance of each experimental procedure involving human subjects should be clearly formulated in an experimental protocol which should be transmitted for consideration, comment and guidance to a specially appointed committee independent of the investigator and the

sponsor provided that this independent committee is in conformity with the laws and regulations of the country in which the research experiment is performed.

3. Biomedical research involving human subjects should be conducted only by scientifically qualified persons and under the supervision of a clinically competent medical person. The responsibility for the human subject must always rest with a medically qualified person and never rest on the subject of the research, even though the subject has given his or her consent.

4. 4. Biomedical research involving human subjects cannot legitimately be carried out unless the importance of the objective is in proportion to the inherent risk to the subject.

5. Every biomedical research project involving human subjects should be preceded by careful assessment of predictable risks in comparison with foreseable benefits to the subject or to others. Concern for the interests of the subject must always prevail over the interests of science and society.

6. The right of the research subject to safeguard his or her integrity must always be respected. Every precaution should be taken to respect the privacy of the subject and to minimize the impact of the study on the subject's physical and mental integrity and on the personality of the subject.

7. Physicians should abstain from engaging in research projects involving human subjects unless they are satisfied that the hazards involved are believed to be predictable. Physicians should cease any investigation if the hazards are found to outweigh the potential benefits.

8. In publication of the results of his or her research, the physician is obliged to preserve the accuracy of the results. Reports of experimentation not in accordance with the principles laid down in this Declaration should not be accepted for publication.

9. In any research on human beings, each potential subject must be adequately informed of the aims, methods, anticipated benefits and potential hazards of the study and the discomfort it may entail. He or she should be informed that he or she is at liberty to abstain from participation in the study and that he or she is free to withdraw his or her consent to participation at any time. The physician should then obtain the subject's freely-given informed consent, preferably in writing.

10. When obtaining informed consent for the research project the physician should be particularly cautious if the subject is in a dependent relationship to him or her or may consent under duress. In that case the informed consent should be obtained by a physician who is not engaged in the investigation and who is completely independent of this official relationship.

11. In case of legal incompetence, informed consent should be obtained from the legal guardian in accordance with national legislation. Where physical or mental incapacity makes it impossible to obtain informed consent, or when the subject is a minor, permission from the responsible relative replaces that of the subject in accordance with national legislation. Whenever the minor child is in fact able to give a consent, the minor's consent must be obtained in addition to the consent of the minor's legal guardian.

12. The research protocol should always contain a statement of the ethical considerations involved and should indicate that the principles enunciated in the present Declaration are complied with.

II. MEDICAL RESEARCH COMBINED WITH PROFESSIONAL CARE (Clinical Research)

1. In the treatment of the sick person, the physician must be free to use a new diagnostic and therapeutic measure, if in his or her judgement it offers hope of saving life, reestablishing health or alleviating suffering.

2. The potential benefits, hazards and discomfort of a new method should be weighed against the advantages of the best current diagnostic and therapeutic methods.
3. In any medical study, every patient - including those of a control group, if any - should be assured of the best proven diagnostic and therapeutic method. This does not exclude the use of inert placebo in studies where no proven diagnostic or therapeutic method exists.
4. The refusal of the patient to participate in a study must never interfere with the physician-patient relationship.
5. If the physician considers it essential not to obtain informed consent, the specific reasons for this proposal should be stated in the experimental protocol for transmission to the independent committee (I, 2).
6. The physician can combine medical research with professional care, the objective being the acquisition of new medical knowledge, only to the extent that medical research is justified by its potential diagnostic or therapeutic value for the patient.

III. NON-THERAPEUTIC BIOMEDICAL RESEARCH INVOLVING HUMAN SUBJECTS (Non-Clinical Biomedical Research)

1. In the purely scientific application of medical research carried out on a human being, it is the duty of the physician to remain the protector of the life and health of that person on whom biomedical research is being carried out.
2. The subject should be volunteers - either healthy persons or patients for whom the experimental design is not related to the patient's illness.
3. The investigator or the investigating team should discontinue the research if in his/her or their judgement it may, if continued, be harmful to the individual.
4. In research on man, the interest of science and society should never take precedence over considerations related to the wellbeing of the subject.

APPENDIX 3 - DEFINITION OF ADVERSE EVENTS

Adverse Event

Any untoward medical occurrence in a patient or clinical trial subject administered a medicinal product and which does not necessarily have a causal relationship with this treatment.

Comment:

An AE can therefore be any unfavourable and unintended sign, symptom or disease temporally associated with the use of an investigational medicinal product, whether or not related to the investigational medicinal product.

Please note this does not include abnormal laboratory findings. An abnormal laboratory value is only considered to be an AE if the abnormality:

- Results in early discontinuation from the study treatment and/or
- Requires study drug dose modification or interruption, any other therapeutic intervention or is judged to be of significant clinical importance

If a laboratory abnormality is one component of a diagnosis or syndrome, then only the diagnosis or syndrome should be recorded.

Adverse Reaction

All untoward and unintended responses to an IMP related to any dose administered.

Comment:

An AE judged by either the reporting Investigator or Sponsor as having causal relationship to the IMP qualifies as an AR. The expression reasonable causal relationship means to convey in general that there is evidence or argument to suggest a causal relationship.

Serious Adverse Event

Any untoward medical occurrence or effect that at any dose:

- Results in death
- Is life-threatening*
- Requires hospitalisation** or prolongation of existing inpatients' hospitalisation
- Results in persistent or significant disability or incapacity
- Is a congenital anomaly/birth defect
- Or is otherwise considered medically significant by the Investigator***

Comments:

The term severe is often used to describe the intensity (severity) of a specific event. This is not the same as serious, which is based on patients/event outcome or action criteria.

* Life threatening in the definition of an SAE refers to an event in which the patient was at risk of death at the time of the event; it does not refer to an event that hypothetically might have caused death if it were more severe.

**Hospitalisation is defined as an unplanned, formal inpatient admission, even if the hospitalisation is a precautionary measure for continued observation. Thus hospitalisation for protocol treatment (e.g. line insertion), elective procedures (unless brought forward because of worsening symptoms) or for social reasons (e.g. respite care) are not regarded as an SAE.

*** Medical judgment should be exercised in deciding whether an AE is serious in other situations. Important AEs that are not immediately life threatening or do not result in death or hospitalisation but may jeopardise the subject or may require intervention to prevent one of the other outcomes listed in the definition above, should be considered serious.

Serious Adverse Reaction

An Adverse Reaction which also meets the definition of a Serious Adverse Event.

Suspected Unexpected Serious Adverse Reaction

A SAR that is unexpected i.e. the nature, or severity of the event is not consistent with the applicable product information.

A SUSAR should meet the definition of an AR, UAR and SAR.

Unexpected Adverse Reaction

An AR, the nature or severity of which is not consistent with the applicable product information (e.g. Investigator Brochure for an unapproved IMP or (compendium of) Summary of Product Characteristics (SPC) for a licensed product).

When the outcome of an AR is not consistent with the applicable product information the AR should be considered unexpected.

APPENDIX 4 - COMMON TOXICITY CRITERIA GRADINGS

Toxicities will be recorded according to the Common Terminology Criteria for Adverse Events (CTCAE), version 4.0. The full CTCAE document is available on the National Cancer Institute (NCI) website, the following address was correct when this version of the protocol was approved:

http://ctep.cancer.gov/protocolDevelopment/electronic_applications/ctc.htm

APPENDIX 5 – EUROPEAN LEUKAEMIANET CRITERIA FOR RESPONSE TO THERAPY IN PV AND ET

The following contain excerpts from the European LeukaemiaNet Criteria.

Ref: Barosi *et al.* (2009). Response criteria for essential thrombocythemia and polycythemia vera: result of a European LeukemiaNet consensus conference. Blood **113** (20) 4829-4833.

These criteria will be used to assess the endpoint of maintenance of haematological response in patients with PV and ET.

Definition of clinicohaematologic response in ET and PV based on European LeukaemiaNet Criteria

	**Definition**	
**Response grade**	**Essential thrombocythemia**	**Polycythaemia vera**
Complete response	(1) Platelet count ≤ 400 x 10^9/L, AND<br>(2) normal spleen size on imaging, AND<br>(3) white blood cell count ≤ 10 x 10^9/L	(1) Haematocrit < 45% without venesection for 3 months AND<br>(2) platelet count ≤ 400 x 10^9/L AND<br>(3) white blood cell count ≤ 10 x 10^9/L, AND<br>(4) normal spleen size on imaging
Partial response	In patients who do not fulfill the criteria for complete response,<br>platelet count ≤ 600 x 10^9/L OR decrease > 50% from baseline	In patients who do not fulfil the criteria for complete response,<br>hematocrit < 45% without venesection for 3 months OR response in 3 of the other criteria
No response	Any response that does not satisfy partial response	Any response that does not satisfy partial response

APPENDIX 6 – REVISED RESPONSE CRITERIA FOR MYELOFIBROSIS

Ref: Tefferi *et al.* (2013). Revised response criteria for myelofibrosis: International Working Group-Myeloproliferative Neoplasms Research and Treatment (IWG-MRT) and European LeukemiaNet (ELN) consensus report. Blood **122** (8) 1395-1398 [2].

These criteria will be used to assess the endpoints of maintenance of response (no evidence of disease progression) in patients with MF and response assessment at 24 weeks.

Response categories	Required criteria (for all response categories, benefit must last for ≥12 wk to qualify as a response)
CR	Bone marrow:* Age-adjusted normocellularity; <5% blasts; ≤grade 1 MF† and<br>Peripheral blood: Hemoglobin ≥100 g/L and <UNL; neutrophil count ≥ 1 x 10^9/L and <UNL;<br>Platelet count ≥100 x 10^9/L and <UNL; <2% immature myeloid cells‡ and<br>Clinical: Resolution of disease symptoms; spleen and liver not palpable; no evidence of EMH
PR	Peripheral blood: Hemoglobin ≥100 g/L and <UNL; neutrophil count ≥1 x 10^9/L and <UNL; platelet count ≥100 x 10^9/L and <UNL; <2% immature myeloid cells‡ and<br>Clinical: Resolution of disease symptoms; spleen and liver not palpable; no evidence of EMH or<br>Bone marrow:* Age-adjusted normocellularity; ,<br><5% blasts; ≤grade 1 MF†, and peripheral blood: Hemoglobin ≥85 but <100 g/L and <UNL; neutrophil count ≥1 x 10^9/L and <UNL; platelet count ≥50, but ,<100 x 10^9/L and <UNL; <2% immature myeloid cells‡ and<br>Clinical: Resolution of disease symptoms; spleen and liver not palpable; no evidence of EMH
Clinical Improvement	The achievement of anemia, spleen or symptoms response without progressive disease or increase in severity of anemia, thrombocytopenia, or neutropenia§
Anaemia Response	Transfusion-independent patients: a ≥20 g/L increase in hemoglobin level|| Transfusion-dependent patients: becoming transfusion-independent¶
Spleen response#	A baseline splenomegaly that is palpable at 5-10 cm, below the LCM, becomes not palpable** or<br>A baseline splenomegaly that is palpable at >10 cm, below the LCM, decreases by ≥50%**<br>A baseline splenomegaly that is palpable at <5 cm, below the LCM, is not eligible for spleen response<br>A spleen response requires confirmation by MRI or computed tomography showing ≥35% spleen volume
Symptoms response	A ≥50% reduction in the MPN-SAF TSS††
Progressive disease	Appearance of a new splenomegaly that is palpable at least 5 cm below the LCM or<br>A ≥100% increase in palpable distance, below LCM, for baseline splenomegaly of 5-10 cm or<br>A 50% increase in palpable distance, below LCM, for baseline splenomegaly of >10 cm or<br>Leukemic transformation confirmed by a bone marrow blast count of ≥20% or<br>A peripheral blood blast content of ≥20% associated with an absolute blast count of ≥1 x 10(9)/L that lasts for at least 2 weeks
Stable disease	Belonging to none of the above listed response categories
Relapse	No longer meeting criteria for at least CI after achieving CR, PR, or CI, or<br>Loss of anemia response persisting for at least 1 month or<br>Loss of spleen response persisting for at least 1 month
Recommendations for assessing treatment-induced cytogenetic and molecular changes	
Cytogenetic remission	At least 10 metaphases must be analyzed for cytogenetic response evaluation and<br>requires confirmation by repeat testing within 6 months window<br>CR: eradication of a pre-existing abnormality<br>PR: ≥50% reduction in abnormal metaphases<br>(partial response applies only to patients with at least ten abnormal metaphases at baseline)
Molecular remission	Molecular response evaluation must be analyzed in peripheral blood granulocytes and<br>requires confirmation by repeat testing within 6 months window<br>CR: Eradication of a pre-existing abnormality<br>PR: ≥50% decrease in allele burden<br>(partial response applies only to patients with at least 20% mutant allele burden at baseline)
Cytogenetic/molecular relapse	Re-emergence of a pre-existing cytogenetic or molecular abnormality that is confirmed by repeat testing

EMH, extramedullary hematopoiesis (no evidence of EMH implies the absence of pathology- or imaging study-proven nonhepatosplenic EMH); LCM, left costal margin; UNL, upper normal limit.
*Baseline and posttreatment bone marrow slides are to be interpreted at one sitting by a central review process. Cytogenetic and molecular responses are not required for CR assignment.
†Grading of MF is according to the European classification
Thiele et al. European consensus on grading bone marrow fibrosis and assessment of cellularity. Haematologica. 2005;90:1128.
It is underscored that the consensus definition of aCR bone marrow is to be used only in those patients in which all other criteria are met, including resolution of leukoerythroblastosis.
It should also be noted that it was a particularly difficult task for the working group to reach a consensus regarding what represents a complete histologic remission.
‡Immature myeloid cells constitute blasts + promyelocytes + myelocytes + metamyelocytes + nucleated red blood cells. In splenectomized patients, <5% immature myeloid cells is allowed.
§See above for definitions of anemia response, spleen response, and progressive disease. Increase in severity of anemia constitutes the occurrence of new transfusion dependency or a ≥20 g/L decrease in hemoglobin level from pretreatment baseline that lasts for at least 12 weeks. Increase in severity of thrombocytopenia or neutropenia is defined as a 2-grade decline, from pretreatment baseline, in platelet count or absolute neutrophil count, according to the Common Terminology Criteria for Adverse Events (CTCAE) version 4.0. In addition, assignment to CI requires a minimum platelet count of ≥25 000 x 10(9)/L and absolute neutrophil count of ≥0.5 x 10(9)/L.
||Applicable only to patients with baseline hemoglobin of <100 g/L. In patients not meeting the strict criteria for transfusion dependency at the time of study enrollment (see as follows), but have received transfusions within the previous month, the pretransfusion hemoglobin level should be used as the baseline.
¶Transfusion dependency before study enrollment is defined as transfusions of at least 6 units of packed red blood cells (PRBC), in the 12 weeks prior to study
enrollment, for a hemoglobin level of <85 g/L, in the absence of bleeding or treatment-induced anemia. In addition, the most recent transfusion episode must have occurred in the 28 days prior to study enrolment. Response in transfusion-dependent patients requires absence of any PRBC transfusions during any consecutive “rolling” 12-week interval during the treatment phase, capped by a hemoglobin level of ≥85 g/L.
#In splenectomized patients, palpable hepatomegaly is substituted with the same measurement strategy.
**Spleen or liver responses must be confirmed by imaging studies where a ≥35% reduction in spleen volume, as assessed by MRI or CT, is required. Furthermore, a ≥35% volume reduction in the spleen or liver, by MRI or CT, constitutes a response regardless of what is reported with physical examination.
††Symptoms are evaluated by the MPN-SAF TSS. The MPN-SAF TSS is assessed by the patients themselves and this includes fatigue, concentration, early satiety, inactivity, night sweats, itching, bone pain, abdominal discomfort, weight loss, and fevers. Scoring is from 0 (absent/as good as it can be) to 10 (worst imaginable/as bad as it can be) for each item. The MPN-SAF TSS is the summation of all the individual scores (0-100 scale). Symptoms response requires ≥50% reduction in the MPN-SAF TSS.
‡‡Progressive disease assignment for splenomegaly requires confirmation my MRI or computed tomography showing a ≥25% increase in spleen volume from baseline.

Baseline values for both physical examination and imaging studies refer to pretreatment baseline and not to posttreatment measurements.

APPENDIX 7 – REVISED RESPONSE CRITERIA FOR POLYCYTHEMIA VERA AND ESSENTIALTHROMBOCYTHEMIA: AN ELN AND IWG-MRT CONSENSUS PROJECT

Ref: Barosi *et al.* (2013). Revised response criteria for polycythemia vera and essential thrombocythemia: an ELN and IWG-MRT consensus project. Blood. **121** (23) 4778-4781.
These criteria will be used to assess the endpoint of response at 24 weeks.

Response criteria for PV

	**Criteria**
**Complete Remission**	
A	Durable* resolution of disease-related signs including palpable hepatosplenomegaly, large symptoms improvement,† AND
B	Durable* peripheral blood count remission, defined as: Ht lower than 45% without phlebotomies; platelet count ≤400 x 10^9/L, WBC count < 10 x 10^9/L, AND
C	Without progressive disease, and absence of any hemorrhagic or thrombotic event, AND
D	Bone marrow histological remission defined as the presence of age-adjusted normocellularity and disappearance of trilinear hyperplasia, and absence of >grade 1 reticulin fibrosis.
Partial remission	
A	Durable* resolution of disease-related signs including palpable hepatosplenomegaly, and large symptoms improvement †, AND
B	Durable* peripheral blood count remission, defined as: Ht lower than 45% without phlebotomies; platelet count ≤400 x 10^9/L, WBC count < 10 x 10^9/L, AND
C	Without progressive disease, and absence of any hemorrhagic or thrombotic event, AND
D	Without bone marrow histological remission, defined as persistence of trilinear hyperplasia.
No response	Any response that does not satisfy partial remission
Progressive disease	Transformation into post-PV myelofibrosis, myelodysplastic syndrome or acute leukemia‡

Molecular response is not required for assignment as complete response or partial response. Molecular response evaluation requires analysis in peripheral blood granulocytes. Complete response is defined as eradication of a pre-existing abnormality. Partial response applies only to patients with at least 20% mutant allele burden at baseline. Partial response is defined as ≥ 50% decrease in allele burden.
WBC, white blood cell.
*Lasting at least 12 wk.
†Large symptom improvement (≥10-point decrease) in MPN-SAF TSS.
‡For the diagnosis of post-PV myelofibrosis, see the IWG-MRT criteria; for the diagnosis of myelodysplastic syndrome and acute leukemia, see WHO criteria.

Response criteria for ET

	Criteria
**Complete Remission**	
A	Durable* resolution of disease-related signs including palpable hepatosplenomegaly, large symptoms improvement,† AND
B	Durable* peripheral blood count remission, defined as: platelet count ≤400 x 10^9/L, WBC count < 10 x 10^9/L, absence of leukoerythroblastosis, AND
C	Without signs of progressive disease, and absence of any hemorrhagic or thrombotic events, AND
D	Bone marrow histological remission defined as disappearance of megakaryocyte hyperplasia and absence of >grade 1 reticulin fibrosis.
Partial remission	
A	Durable* resolution of disease-related signs including palpable hepatosplenomegaly, and large symptoms improvement, AND
B	Durable* peripheral blood count remission, defined as: platelet count ≤400 x 10^9/L, WBC count , < 10 x 10^9/L, absence of leukoerythroblastosis, AND
C	Without signs of progressive disease, and absence of any hemorrhagic or thrombotic events, AND
D	Without bone marrow histological remission, defined as the persistence of megakaryocyte hyperplasia.
No response	Any response that does not satisfy partial remission
Progressive disease	Transformation into PV, post-ET myelofibrosis, myelodysplastic syndrome or acute leukemia‡

Molecular response is not required for assignment as complete response or partial response. Molecular response evaluation requires analysis in peripheral blood granulocytes. Complete response is defined as eradication of a pre-existing abnormality. Partial response applies only to patients with at least 20% mutant allele burden at baseline. Partial response is defined as ≥50% decrease in allele burden.

WBC, white blood cell.

*Lasting at least 12 wk.

†Large symptom improvement (≥10-point decrease) in MPN-SAF TSS.

‡For the diagnosis of PV see World Health Organization criteria (WHO); for the diagnosis of post-ET myelofibrosis, see the IWG-MRT criteria; for the diagnosis of myelodysplastic syndrome and acute leukemia, see WHO criteria.

APPENDIX 8 – LIST OF PROHIBITED CONCOMITANT MEDICATION AND LINK TO TAMOXIFEN SPC

At the time of writing, the SPC for tamoxifen can be found using the following link: https://www.medicines.org.uk/emc/medicine/30769

Potent inhibitors of CYP2D6 (e.g. paroxetine, fluoxetine, quinidine, cinacalcet or bupropion) should whenever possible be avoided during tamoxifen treatment.

As tamoxifen is metabolised by CYP3A4, care is required when co-administering with drugs, such as rifampicin, known to induce this enzyme as tamoxifen levels may be reduced.

APPENDIX 9 – MPN SYMPTOM ASSESSMENT FORM TOTAL SYMPTOM SCORE (MPN-SAF TSS)

Myeloproliferative Neoplasm Symptom Assessment Form Total Symptom Score (MPN-SAF TSS)

**Symptom**	**1 to 10 (0 if absent) ranking (1 is most favorable and 10 least favorable)**
Please rate your fatigue (weariness, tiredness) by circling the one number that best describes your WORST level of fatigue during the past **24 hours**	(No Fatigue) 0 1 2 3 4 5 6 7 8 9 10 (Worst Imaginable)
**Circle the one number that describes how, during the PAST 24 HOURS how much difficulty you have had with each of the following symptoms**	
Filling up quickly when you eat (Early Satiety)	(Absent) 0 1 2 3 4 5 6 7 8 9 10 (Worst Imaginable)
Abdominal discomfort	(Absent) 0 1 2 3 4 5 6 7 8 9 10 (Worst Imaginable)
Inactivity	(Absent) 0 1 2 3 4 5 6 7 8 9 10 (Worst Imaginable)
Problems with Concentration - Compared to prior to my MPD	(Absent) 0 1 2 3 4 5 6 7 8 9 10 (Worst Imaginable)
Night Sweats	(Absent) 0 1 2 3 4 5 6 7 8 9 10 (Worst Imaginable)
Itching (pruritus)	(Absent) 0 1 2 3 4 5 6 7 8 9 10 (Worst Imaginable)
Bone Pain (diffuse not joint pain or arthritis)	(Absent) 0 1 2 3 4 5 6 7 8 9 10 (Worst Imaginable)
Fever (>100 F)	(Absent) 0 1 2 3 4 5 6 7 8 9 10 (Daily)
Unintentional weight loss last 6 months	(Absent) 0 1 2 3 4 5 6 7 8 9 10 (Worst Imaginable)
What is your Overall Quality of Life?	(As good as it can be) 0 1 2 3 4 5 6 7 8 9 10 (As bad as it can be)

Trials Office Contact Details

TAMARIN Trial Office
Cancer Research UK Clinical Trials Unit (CRCTU)
Centre for Clinical Haematology
Queen Elizabeth Hospital
Edgbaston, Birmingham, B15 2TH
Tel: 0121 371 7866
Fax: 0121 371 7874
Email: TAMARIN@trials.bham.ac.uk
