# Supplementary material for: Tamoxifen for the treatment of myeloproliferative neoplasms: A Phase II clinical trial and exploratory analysis
Source: Nat Commun. 2023 Nov 25;14:7725. doi: 10.1038/s41467-023-43175-5 (PMC10673935; doi:10.1038/s41467-023-43175-5)
Supplement: Supplementary file 2 — Reporting Summary [file 41467_2023_43175_MOESM2_ESM.pdf]

nature portfolio

Corresponding author(s): Claire N Harrison
Simón Méndez-Ferrer

Last updated by author(s): Oct 5, 2023

Reporting Summary

Nature Portfolio wishes to improve the reproducibility of the work that we publish. This form provides structure for consistency and transparency in reporting. For further information on Nature Portfolio policies, see our Editorial Policies and the Editorial Policy Checklist.

Statistics

For all statistical analyses, confirm that the following items are present in the figure legend, table legend, main text, or Methods section.

n/a	Confirmed	
☐	☒	The exact sample size (n) for each experimental group/condition, given as a discrete number and unit of measurement
☐	☒	A statement on whether measurements were taken from distinct samples or whether the same sample was measured repeatedly
☐	☒	The statistical test(s) used AND whether they are one- or two-sided<br>*Only common tests should be described solely by name; describe more complex techniques in the Methods section.*
☐	☒	A description of all covariates tested
☐	☒	A description of any assumptions or corrections, such as tests of normality and adjustment for multiple comparisons
☐	☒	A full description of the statistical parameters including central tendency (e.g. means) or other basic estimates (e.g. regression coefficient) AND variation (e.g. standard deviation) or associated estimates of uncertainty (e.g. confidence intervals)
☐	☒	For null hypothesis testing, the test statistic (e.g. F, t, r) with confidence intervals, effect sizes, degrees of freedom and P value noted<br>*Give P values as exact values whenever suitable.*
☐	☒	For Bayesian analysis, information on the choice of priors and Markov chain Monte Carlo settings
☐	☒	For hierarchical and complex designs, identification of the appropriate level for tests and full reporting of outcomes
☒	☐	Estimates of effect sizes (e.g. Cohen's d, Pearson's r), indicating how they were calculated

Our web collection on statistics for biologists contains articles on many of the points above.

Software and code

Policy information about availability of computer code

Data collection	No software was used for data collection.
Data analysis	In seahorse metabolic assay, data analysis was performed using the Seahorse XFe Wave software and statistics were analysed in Prism9. Flow cytometry were analysed with a Gallios Flow Cytometer using Kaluza software (BD Biosciences) and statistics were analysed in Prism. Sequencing raw data was analyzed using Casava v1.8.2 pipeline, cutadapt software tool (MIT), RSEM v1.17 and TopHat2. Differential analysis was done by DeSeq2, and gene set enrichment analysis was performed in GSEA software (Broad Institute). Immunological staining was quantified by Fiji ImageJ. Confocal image was visualized by LSM 980 Airyscan 2 (Zeiss). Colocalization analysis was performed by ImageJ plugin Colocalization Finder.

For manuscripts utilizing custom algorithms or software that are central to the research but not yet described in published literature, software must be made available to editors and reviewers. We strongly encourage code deposition in a community repository (e.g. GitHub). See the Nature Portfolio guidelines for submitting code & software for further information.

Data

Policy information about availability of data

All manuscripts must include a data availability statement. This statement should provide the following information, where applicable:

- Accession codes, unique identifiers, or web links for publicly available datasets
- A description of any restrictions on data availability
- For clinical datasets or third party data, please ensure that the statement adheres to our policy

Datasets (raw data) underlying the figures have been provided as source data.
No custom code was used in this study.
The Clinical Study Protocol and the Statistical Analysis Plan, including the sample size calculations, interim safety analysis, analysis methods, Bayesian analysis, hematological response and other exploratory outcomes are available online. For clinical trial data, the detailed information will be available in study report.
The RNAseq data from CD34+ HSPCs of Study subjects has been deposited in GEO under the Accession Number GSE172022. GSE172022 (https://www.ncbi.nlm.nih.gov/geo/query/acc.cgi?acc=GSE172022).
The RNAseq data from HEL cells and UKE-1 cells has been deposited in GEO under the Accession Number GSE172023 (https://www.ncbi.nlm.nih.gov/geo/query/acc.cgi?acc=GSE172023).

Research involving human participants, their data, or biological material

Policy information about studies with human participants or human data. See also policy information about sex, gender (identity/presentation), and sexual orientation and race, ethnicity and racism.

Reporting on sex and gender	Sex-based analysis was included in subgroup analysis of clinical study report.
Reporting on race, ethnicity, or other socially relevant groupings	The information of socially relevant groups was not relevant in clinical study.
Population characteristics	Eligibility criteria included MPN patients aged ≥60 years (50-87-year old), with eligible women being post-menopausal (defined as amenorrhoeic for at least 12 consecutive months following cessation of all exogenous hormonal treatments); confirmed diagnosis of JAK2-V617F, CALR 5bp insertion (exon 9), or CALR 52bp deletion (exon 9) positive ET, PV or MF (primary or secondary) for ≥ 6 months. JAK2-V617F, CALR 5bp insertion (exon 9), or CALR 52bp deletion (exon 9) mutant allele burden ≥20% in peripheral blood granulocyte DNA at study entry (assessed via central review); WHO performance status 0-2. For patients with PV or ET, maintenance of platelet count ≤600 x 109/L, WBC ≤25 ×109/L and venesection requirements ≤1 per month for the previous 3 months prior to registration, without introduction of any new therapeutic agents for their MPN for 6 months prior to registration. For patients with MF, eligible patients showed no evidence of disease progression defined by IWG-MRT ELN criteria or new therapeutic agents introduced for a 6-month period before registration. Patients receiving cytoreductive therapy (with the exception of interferon alpha or investigational agents) for their MPN (not solely aspirin or venesection) were included, and this therapy was continued throughout. Adequate hepatic function, defined as bilirubin ≤1.5 × upper limit of normal (ULN) (patients with elevated bilirubin due to Gilbert's syndrome were eligible) or AST/ALT/ALP ≤2.5 x ULN, and adequate renal function (creatinine clearance >30 mL/min) were required. Male patients agreed to use effective contraception during participation in the trial and for 2 months after the last dose of trial treatment. All patients provided written informed consent.
Recruitment	A total of 38 patients, comprising of 27 males and 11 females, were recruited to the trial over 824 days (118 weeks). The first patient was recruited on 15-Feb-2017, and the last one on 20-May-2019.
Ethics oversight	The study was approved by NHS Health Research Authority (IRAS 201126). The study was conducted in accordance with the Declaration of Helsinki and UK regulations (The Medicines for Human Use (Clinical Trials) Regulations 2004). The clinical trial authorization was provided by the Medicines and Healthcare products Regulatory Agency.

Note that full information on the approval of the study protocol must also be provided in the manuscript.

Field-specific reporting

Please select the one below that is the best fit for your research. If you are not sure, read the appropriate sections before making your selection.

☒ Life sciences ☐ Behavioural & social sciences ☐ Ecological, evolutionary & environmental sciences

For a reference copy of the document with all sections, see nature.com/documents/nr-reporting-summary-flat.pdf

Life sciences study design

All studies must disclose on these points even when the disclosure is negative.

Sample size	No sample size estimation was done for in vitro cell experiments. In general, at least 3 biological replicates were performed from at least three independent experiments. The TAMARIN trial uses an A'hern's design. It was not anticipated that any patients would achieve reduction of 50% or more in allele burden on their current treatment. Therefore, if 10% of patients were to experience such a

	reduction, the treatment would warrant further investigation. If only 2% of patients achieved the primary outcome, then tamoxifen would not be considered to show sufficient activity to be investigated further. Using a one sided alpha of 0.1 and 80% power, 42 patients would need to be recruited to the trial. Under this design, for treatment to be classed successful, 3 patients would need to attain the necessary decrease.
Data exclusions	No data were excluded from the analysis of cell experiments, with the exception of seahorse metabolic assay, where the replicate was excluded if the measurement did not fall within the optimal detection range in Seahorse Real-Time Cell Metabolic Analysis.
Replication	For experiments using cells, at least three independent experiments were performed for the functional assay. Three biological replicates were prepared for RNAseq analysis. No data were excluded from the analyses. Consistent results were obtained using at least three biological replicates. All attempts at replication were successful.
Randomization	For cell culture experiment, cells were equally distributed into the wells of the same plate in each biological replicate and the treatment condition was randomly applied. In the study, experiments were done pair-wise with treated cells/tissue being directly compared to untreated cells/tissue.
Blinding	The investigators were not blinded to allocation during in vitro or animal experiments and outcome assessment.

Reporting for specific materials, systems and methods

We require information from authors about some types of materials, experimental systems and methods used in many studies. Here, indicate whether each material, system or method listed is relevant to your study. If you are not sure if a list item applies to your research, read the appropriate section before selecting a response.

Materials & experimental systems

n/a	Involved in the study
	☒ Antibodies
	☒ Eukaryotic cell lines
☒	☐ Palaeontology and archaeology
	☒ Animals and other organisms
	☒ Clinical data
☒	☐ Dual use research of concern
☒	☐ Plants

Methods

n/a	Involved in the study
☒	☐ ChIP-seq
	☒ Flow cytometry
☒	☐ MRI-based neuroimaging

Antibodies

Antibodies used	FITC Annexin V antibody (BioLegend, Cat. No. 640906, RRID: AB_2561292)<br>anti–pY694-STAT5 (BD Biosciences, clone 47, Cat. No. 562076, RRID: AB_11154412)<br>isotype control (BD Biosciences, clone MOPC-21, Cat. No. 557732, RRID: AB_396840)<br>ERα Antibody (Santa Cruz, Cat. No. sc-8002, clone F-10, RRID: AB_627558)<br>Phospho-eIF2α XP antibody (Ser51) (Cell Signaling Technology, Cat. No. 3398, clone D9G8, RRID: AB_2096481)<br>eIF2α antibody (Cell Signaling Technology, Cat. No. 5324, clone D7D3, RRID: AB_10692650)<br>ATF-4 antibody (Cell Signaling Technology, Cat. No. 11815, clone D4B8, RRID: AB_2616025)<br>Phospho-Stat5 Antibody (Thermo Fisher Scientific, Cat. No. 71-6900, RRID: AB_2533991)<br>Stat5 antibody (BD Biosciences, Cat. No. 610191, RRID: AB_397590)<br>JAK2 antibody (Thermo Fisher Scientific, Cat. No. AHO1352, clone 691R5, RRID: AB_2536334)<br>Phospho-JAK2 (Tyr1007/1008) Antibody (Cell Signaling Technology, Cat. No. 3771, RRID: AB_330403)<br>Phospho-JAK2 (Tyr221) Antibody (Cell Signaling Technology, Cat. No. 3774, RRID: AB_390750)<br>Anti-VDAC1/Porin Antibody (Santa Cruz, Cat. No. sc-390996, clone B-6, RRID: AB_2750920)<br>α-Tubulin antibody (Sigma-Aldrich, Cat. No. T5168, clone B-5-1-2, RRID:AB_477579)<br>TOM20 antibody (Santa Cruz, Cat. No. sc-17764, clone F-10, RRID: AB_628381)<br>Goat anti-Rabbit IgG (H+L) Secondary Antibody, HRP (Invitrogen, Cat. No. 31460, RRID:AB_228341)<br>Goat Anti-Mouse IgG H&L (HRP) (abcam, Cat. No. ab97023, RRID:AB_10679675)
Validation	All commercial antibodies were validated by their manufacturers and were titrated in the lab to determine optimal concentration for experimentation as indicated on manufacturer website (see below).<br>FITC Annexin V antibody: https://www.biolegend.com/fr-ch/products/fitc-annexin-v-5161?GroupID=BLG6046<br>anti–pY694-STAT5: https://www.bdbiosciences.com/en-gb/products/reagents/flow-cytometry-reagents/research-reagents/single-color-antibodies-ruo/alexa-fluor-647-mouse-anti-stat5-py694.562076<br>isotype control: https://www.bdbiosciences.com/en-gb/products/reagents/flow-cytometry-reagents/research-reagents/flow-cytometry-controls-and-lysates/alexa-fluor-647-mouse-igg1-isotype-control.557732<br>ERα Antibody: https://www.scbt.com/p/estrogen-receptor-alpha-antibody-f-10<br>Phospho-eIF2α XP antibody (Ser51): https://www.cellsignal.com/products/primary-antibodies/phospho-eif2a-ser51-d9g8-xp-rabbit-mab/3398?_requestid=5687224<br>eIF2α antibody: https://www.cellsignal.com/products/primary-antibodies/eif2a-d7d3-xp-rabbit-mab/5324

ATF-4 antibody: https://www.cellsignal.com/products/primary-antibodies/atf-4-d4b8-rabbit-mab/11815
Phospho-Stat5 Antibody: https://www.thermofisher.com/antibody/product/Phospho-STAT5-alpha-Tyr694-Antibody-Polyclonal/71-6900
Stat5 antibody: https://www.bdbiosciences.com/en-us/products/reagents/microscopy-imaging-reagents/immunofluorescence-reagents/purified-mouse-anti-stat5.610192
JAK2 antibody: https://www.thermofisher.com/antibody/product/JAK2-Antibody-clone-691R5-Monoclonal/AHO1352
Phospho-JAK2 (Tyr1007/1008) Antibody: https://www.cellsignal.com/products/primary-antibodies/phospho-jak2-tyr1007-1008-antibody/3771
https://www.sigmaaldrich.com/GB/en/product/sigma/t5168
Phospho-JAK2 (Tyr221) Antibody: https://www.cellsignal.com/products/primary-antibodies/phospho-jak2-tyr221-antibody/3774
Anti-VDAC1/Porin Antibody: https://www.scbt.com/p/vdac1-antibody-b-6
α-Tubulin antibody:https://www.sigmaaldrich.com/GB/en/product/sigma/t5168
TOM20 antibody: https://www.scbt.com/p/tom20-antibody-f-10

Eukaryotic cell lines

Policy information about cell lines and Sex and Gender in Research

Cell line source(s)	Human MPN cell lines HEL 92.1.7 (CVCL_2481), UKE-1 (CVCL_0104) and SET-2 (CVCL_2187), mouse pro-B cell line Ba/F3 (CVCL_0161) and human embryonic kidney HEK293T (CVCL_0063) were obtained from the American Type Culture Collection (ATCC; http://www.atcc.org, Manassas, VA). The human oestrogen receptor-positive breast cancer MCF-7 were provided by Dr Jason Carroll (Cancer Research UK).The MARIMO cell line was kindly provided by Dr Juan Li (Wellcome-MRC Cambridge Stem Cell Institute)
Authentication	Cell lines were authenticated by supplier. None of these cell lines were authenticated in house for this manuscript.
Mycoplasma contamination	Mycoplasma Free
Commonly misidentified lines (See ICLAC register)	No commonly misidentified cell lines were used in this study.

Animals and other research organisms

Policy information about studies involving animals; ARRIVE guidelines recommended for reporting animal research, and Sex and Gender in Research

Laboratory animals	Age-matched, wild-type C57BL/6J mice (Charles River Laboratories) were used for in vivo treatments and as recipients of bone marrow transplantation assays to generate MPN model. Mice were culled at 20-23 weeks when the MPN phenotype developed. . Mice were housed in specific pathogen-free facilities, with relative humidity at 45 to 65%, and temperature at 20-24 oC. The house mice under 12:12 light/dark cycles.
Wild animals	No wild animal was used in this study.
Reporting on sex	Only female C57BL/6J mice were used in this study.
Field-collected samples	No field-collected sample was used in this study.
Ethics oversight	All experiments using mice followed protocols approved by the Animal Welfare Ethical Committees (AWERB) at the University of Cambridge (PPL P0242B783). All experiments were compliant with UK and EU recommendations.

Note that full information on the approval of the study protocol must also be provided in the manuscript.

Clinical data

Policy information about clinical studies
All manuscripts should comply with the ICMJEguidelines for publication of clinical research and a completedCONSORT checklist must be included with all submissions.

Clinical trial registration	EudraCT Number: 2015-005497-38
Study protocol	Tamoxifen was provided at the common dose used in ER+ breast cancer (20 mg oral daily, progressively escalated to 40 mg daily upon good tolerance and when there was no hematological response or mutant allele burden reduction at 12w). All patients received trial treatment for 24 weeks. Treatment continuation was encouraged but not mandated after 24w for patients who did not experience persistent side effects greater than grade 1 or thrombotic events of any grade and that fulfilled one or more of the following criteria at 24 weeks: ≥25% reduction in allele burden compared to baseline; improvement of hematological response compared to baseline without changes in cytoreductive therapy dose according to 2009 ELN criteria for ET/PV patients and to IWG-MRT response criteria for MF patients; a decrease in requirement for cytoreduction without deterioration of hematological response compared to baseline according to 2009 ELN criteria for ET/PV patients and to IWG-MRT response criteria for MF patients. Their response was reassessed after 36 and 48 weeks of treatment as applicable. The Study Protocol is available in the Supplementary Information.
Data collection	A total of 38 patients, comprising of 27 males and 11 females, were recruited to the trial over 824 days (118 weeks).

	The first patient was recruited on 15-Feb-2017, and the last one on 20-May-2019. This trial used an electronic remote data capture (eRDC) system for completion of CRFs. Access to the eRDC system was granted to individuals via the Trials Office. SAE reporting and Notification of Pregnancy were paper-based. The CRF was completed by the Investigator or an authorized member of the site research team (as delegated on the Site Signature and Delegation Log).
Outcomes	The primary endpoint was a reduction in the peripheral blood JAK2V617F, CALR 5bp insertion (exon 9) or CALR 52bp deletion (exon 9) mutant allele burden of ≥50% at 24 weeks. Under the A'hern design, it was necessary to observe at least 3 successes (i.e. reductions in allele burden of ≥50% in the 38 patients recruited). Secondary outcomes were the proportion of patients with a reduction in the peripheral blood JAK2-V617F, CALR 5bp insertion (exon 9), or CALR 52bp deletion (exon 9) mutant allele burden of ≥50% at 12 weeks; toxicity measured as the number of grade 3 and 4 adverse events reported; the number of thrombotic events of any grade reported and validated; duration of hematological response calculated as time from registration to progression for patients who entered the study in response (CR or PR). For patients who entered the trial in stable disease, the time between first recorded response to the date of progression. Progression was defined as loss of response for PV/ET patients and evidence of disease progression for MF patients. PV/ET patients who continued to achieve a response, or MF patients who had no evidence of disease progression at the end of the trial were censored at date last seen. Hematological response was defined according to 2009 ELN criteria for ET/PV patients and no evidence of disease progression for MF patients according to IWG-MRT response criteria ; proportion of patients in each response category according to IWG-MRT response criteria for MF patients and 2013 ELN response criteria for ET/PV patients at 24 weeks of treatment; proportion of patients showing an improvement in response category at 24 weeks compared to baseline according to 2009 ELN criteria for ET/PV patients and according to IWG-MRT response criteria for MF patients; patients who are in a higher category at week 24 compared to baseline were classed a success; patients who enter the trial in CR and who maintain a CR were classed as a success in this outcome. Exploratory outcomes included the proportion of patients showing a decrease in allele burden at 12 and 24 weeks compared to baseline was presented as the number of patients who have shown a decrease of any amount between baseline and week 12 or between baseline and week 24; proportion of patients showing a decrease in requirement for cytoreduction therapy at 24 weeks compared to baseline; proportion of patients showing a decrease in allele burden of ≥50% at 36 and 48 weeks compared to baseline; duration of reduction in the peripheral blood JAK2-V617F, CALR 5bp insertion (exon 9), or CALR 52bp deletion (exon 9) mutant allele burden, defined as time from first observed reduction of ≥50% until reduction from baseline becomes <25%; RNAseq studies on CD34+ HSPCs isolated from peripheral blood at different time points throughout the study.

Flow Cytometry

Plots

Confirm that:

- [x] The axis labels state the marker and fluorochrome used (e.g. CD4-FITC).
- [x] The axis scales are clearly visible. Include numbers along axes only for bottom left plot of group (a 'group' is an analysis of identical markers).
- [x] All plots are contour plots with outliers or pseudocolor plots.
- [x] A numerical value for number of cells or percentage (with statistics) is provided.

Methodology

Sample preparation	For measurement of pSTAT5, cells were washed with PBS and fixed in 2% paraformaldehyde at 37°C for 10 minutes, centrifuged, washed once in p-STAT staining buffer (PBS, pH 7.2, with 0.2% BSA and 0.09% sodium azide) and permeabilized in 100% ice cold methanol on ice for 30 minutes. Cells were washed twice with pSTAT staining buffer and labelled with anti–pY694-STAT5 (clone 47, BD Biosciences) or isotype control for 20 minutes. Cells were washed once in pSTAT staining buffer.<br><br>To measure apoptosis, cells were harvested after 24h and 48h incubation, washed with Annexin V binding buffer (140 mM NaCl, 4 mM KCl, 0.75 mM MgCl2 and 10 mM HEPES in bidistilled water, pH 7.4) and labelled using the FITC Annexin V antibody (catalog number 640906, BioLegend UK) and DAPI.<br><br>To measure the ATF4 mScarlet level, cells were washed by PBS and stained with DAPI before loading.
Instrument	Gallios Flow Cytometer (Beckman Coulter)
Software	Kaluza (Flow Cytometry Analysis Software)
Cell population abundance	To measure pSTAT5 level, all singlet events were analyzed. (no post-sort fractioning was performed in this experiment).<br>To measure apoptosis, the fraction of live cells was determined by selecting Annexin- DAPI- cells.<br>To measure ATF4 mScarlet level, the reporter fluorescence level was determined by selecting DAPI- cells.
Gating strategy	pSTAT5 level was measured on Channel 6 (660 BP 20)<br>FITC-Annexin V was measured on Channel 1 (525 BP)<br>DAPI was measured on Channel 9 (450 BP 50)<br>ATF4 mScarlet was measured on Channel 3 (620 BP 30)

- [x] Tick this box to confirm that a figure exemplifying the gating strategy is provided in the Supplementary Information.
