# Supplementary material for: Tamoxifen for the treatment of myeloproliferative neoplasms: A Phase II clinical trial and exploratory analysis
Source: Nat Commun. 2023 Nov 25;14:7725. doi: 10.1038/s41467-023-43175-5 (PMC10673935; doi:10.1038/s41467-023-43175-5)
Supplement: Supplementary file 3 — Description of Additional Supplementary Files [file 41467_2023_43175_MOESM3_ESM.pdf]

Title: Supplementary Data 1

Description: Study subjects, demographics, responses and allele burden changes. Disease subtype, driver mutation, baseline allele burden and allele burden change (%) 12w and 24w after tamoxifen treatment in 38 Study subjects (each row is an individual). Shaded colors indicate allele burden reduction at 24w (green, ≥50%; yellow, ≥25, <50%; blush, <25%). ET, essential thrombocythemia. PV, polycythemia vera. MF, myelofibrosis. PMF, primary MF.

Title: Supplementary Data 2

Description: Comparison of HSPC transcriptome between tamoxifen responders and non-responders at baseline. Sample information of study subjects at baseline, matrix of raw gene counts, normalized gene counts, differential gene expression analysis (Gene_DE_baseline), GO terms enriched in responders and non-responders (GO analysis results), and gene set enrichment analysis (GSEA) showing pathways enriched in responders or non-responders, or comparing baseline expression in responders reaching ≥50% reduction (major) or ≥25-50% (minor) allele burden reductions after 24w tamoxifen treatment.

Title: Supplementary Data 3

Description: RNA-Seq analysis of human *JAK2*$^{V617F+}$ cell lines before and after 4OH-TAM treatment. Sample information, matrix of raw gene counts, normalized gene counts and gene set enrichment analysis (GSEA). To interrogate differentiated-expressed (DE) genes in each cell line (HEL, UKE-1 in competent medium and UKE-1 in horse serum-free medium), pairwise comparisons between control and 4OH-TAM treatment were performed, following by GSEA using top DE genes (FDR<0.01). The positive and negative normalized enrichment score (NES) indicate gene sets enriched in control group or 4OH-TAM-treated cells, respectively. The genes that are differentially expressed in tamoxifen-sensitive cell lines only are collected in “Pre-ranked Genelist HEL” and “Pre-ranked Genelist UKE-1-HS” with their specific expression levels in the two cell lines. Gene sets enriched in the control group or in 4OH-TAM-treated cells using pre-ranked GSEA are collected in “GSEA pre-ranked HEL” and “GSEA pre-ranked UKE-1-HS”.

Title: Supplementary Data 4

Description: Analysis of HSPC transcriptome in tamoxifen responders and non-responders at baseline and after 24w tamoxifen treatment. Sample information of study subjects, matrix of raw gene counts, normalized gene counts, and GSEA of HPSC transcriptome from responders and non-responders before treatment and after 24w tamoxifen treatment. Gene sets enriched in pre- and post-treatment groups of responder and non-responder study subjects are listed.
